# Transition of a small Himalayan glacier lake outburst flood to a giant transborder flood and debris flow

**DOI:** 10.1038/s41598-022-16337-6

**Published:** 2022-07-20

**Authors:** Ashim Sattar, Umesh K. Haritashya, Jeffrey S. Kargel, Alina Karki

**Affiliations:** 1 Department of Geology and Environmental Geosciences, University of Dayton, Dayton, OH 45469 USA; 2 Environment and Climate: Impacts, Risks and Adaptation (EClim), Department of Geography, University of Zurich, Zurich, Switzerland; 3 Planetary Science Institute, Tucson, AZ 85719 USA; 4 Nepal Electricity Authority, Kathmandu, Nepal

**Keywords:** Hydrology, Natural hazards

## Abstract

Glacial lake outburst floods (GLOFs) are a great concern for the Himalaya, as they can severely damage downstream populations and infrastructures. These floods originate at high altitudes and can flow down with enormous energy and change the terrain’s existing morphology. One such devastating event occurred on the night of 5 July 2016, from the inconspicuous Gongbatongsha Lake, located in the Poiqu basin, Eastern Himalaya. The Poiqu basin in the Tibetan Autonomous Region currently contains numerous big glacial lakes; however, this event originated from a small lake. The GLOF was triggered following heavy precipitation that led to a slope failure above the lake and deposition of debris into the lake, which breached the moraine dam and rapidly drained the entire lake. The flood damaged several downstream infrastructures, including the Arniko highway, the Upper Bhotekoshi hydropower plant, and several buildings as it made its way into the Bhotekoshi basin in Nepal. This study adopts a multi-model approach to reconstruct the GLOF trigger and the flood’s transformation into a severe debris flow. Proxies including flow discharge, flow velocity, runout distances were used to calibrate the model and validate the results. Results reveal that a debris flow of volume ranging between 3000 and 6000 m^3^ from the headwall must have led to lake overfill, eventually leading to the GLOF event. The GLOF showed a significant increase in peak discharge from 618 to 4123 m^3^ s^−1^ at the Zhangzangbo-Bhotekoshi confluence. The average velocity of the flow is calculated to be ~ 5.5 m s^−1^. Reconstruction of the erosion and deposition dynamics show that maximum erosion occurred in the first 6.5 km, with maximum deposition occurring near the Upper Bhotekoshi hydropower station. The modeling indicates that the availability of the entrainable debris along the channel, likely from the previous landslides, amplified the event by three orders of magnitude-additional water ingested from the river. Overall, we demonstrate how the small-scale Gongbatongsha GLOF amplified downstream by incorporating pre-existing sediment in the valley and triggered damaging secondary landslides leading to an economic loss of > 70 million USD.

## Introduction

Climate change-driven glacier retreat leads to the formation of numerous glacial lakes in the Himalaya^1–4^. Of particular concern are the floods that originate from these lakes due to catastrophic failure of damming moraine, most often triggered by an impact from mass wasting entering the lake or sediment overfilling the lake^4–6^. These Glacial Lake Outburst Floods (GLOFs) possess great potential to cause far-reaching damage to the downstream regions^3,7,8^. A recent study shows that GLOFs are expected to increase in the coming decades, the occurrence of which has been fluctuating in the past, rising initially starting in the 1930s and then falling after the 1980s^9,10^. The mid-twentieth century spike has been interpreted as a delayed response to the end of the Little Ice Age and the subsequent decline as a delayed response to climate stabilization after the Little Ice Age^9^. Considering multi-decadal or century-long response times of glaciers and glacial lakes, an increase in GLOFs in the twenty-first century due to anthropogenic warming is projected^9^.

The eastern Himalaya is currently identified as a dangerous hotspot of GLOF hazards, which are likely to spread or shift westwardly in the Himalaya in the future^11^, posing both hazards and risks. GLOF hazards entail a complex interplay of geological forces with the long-term climate trends and extreme weather action on the Earth surface system, while GLOF risks involve a further interaction of the changing hazards with the built and populated environment^12,13^, which, as in other places, is rapidly developing in the Himalaya. Hence, hazards, exposure, and vulnerability increase and aggravate the Himalayan GLOF risk^14^.

The Tibetan Plateau (TP) is home to numerous hazardous lakes, of which at least 40 lakes have witnessed a GLOF in the past^15,16^. Many of the existing lakes in the TP pose transboundary threats downstream^12,16,17^. The Poiqu/Bhotekoshi basin is one such basin in which the majority area lies in the TP and the lower portion lies in Nepal. The basin currently hosts numerous hazardous lakes^15,16,18,19^ and has been extensively studied with a focus on glacial lake changes and GLOF hazard potential^12,15,20,21^. The glaciers in the basin show mass loss and accelerated retreat after 2000^21–23^. Consequently, there has been the formation of high-altitude lakes and an increase in the size of the existing ones^1,19–21^. Of the total of 122 glacial lakes (> 0.01 km^2^) in the basin, different studies have identified different sets of the lake as potentially dangerous. For example, a 2019 study identified 8 lakes as potentially dangerous^21^; another 2019 study identified 7 potentially dangerous lakes with transboundary threats along the China-Nepal border^16^; a 2020 study identified twenty glacial lakes as very highly or highly hazardous^19^. Furthermore, any one of several lakes in the valley, even if they emit a flood at the lowest considered discharge, could cause immense damage in China and across the border into Nepal^24^, as we report in this study.

We note that geomorphological evidence shows a long history of GLOFs in the Zhangzangbo valley^24^ (a part of the Poiqu/Bhotekoshi basin), some of which have caused a severe impact^12^. Taraco Lake, a moraine-dammed lake located in the Tajilingpu valley, had an outburst in August 1935 where livestock and 66,000 m^2^ of agricultural fields were destroyed^20^. In June 2002, two large debris flow resulting from the Jailonngco Lake’s moraine failure occurred in the Chongdui valley and damaged the China-Nepal highway and the hydropower station, leading to an economic loss of over a million dollars^20^. The Cirenmaco lake, located in the Zhangzangbo valley, witnessed three major GLOFs in 1964, 1981, and 1983^25^. Of the three events, the 1981 flood was the most catastrophic that killed over 200 people, destroyed the Friendship Bridge, and severely affected the Sunkoshi Hydropower station in Nepal^24–26^.

### The 2016 GLOF

**Figure 1:**
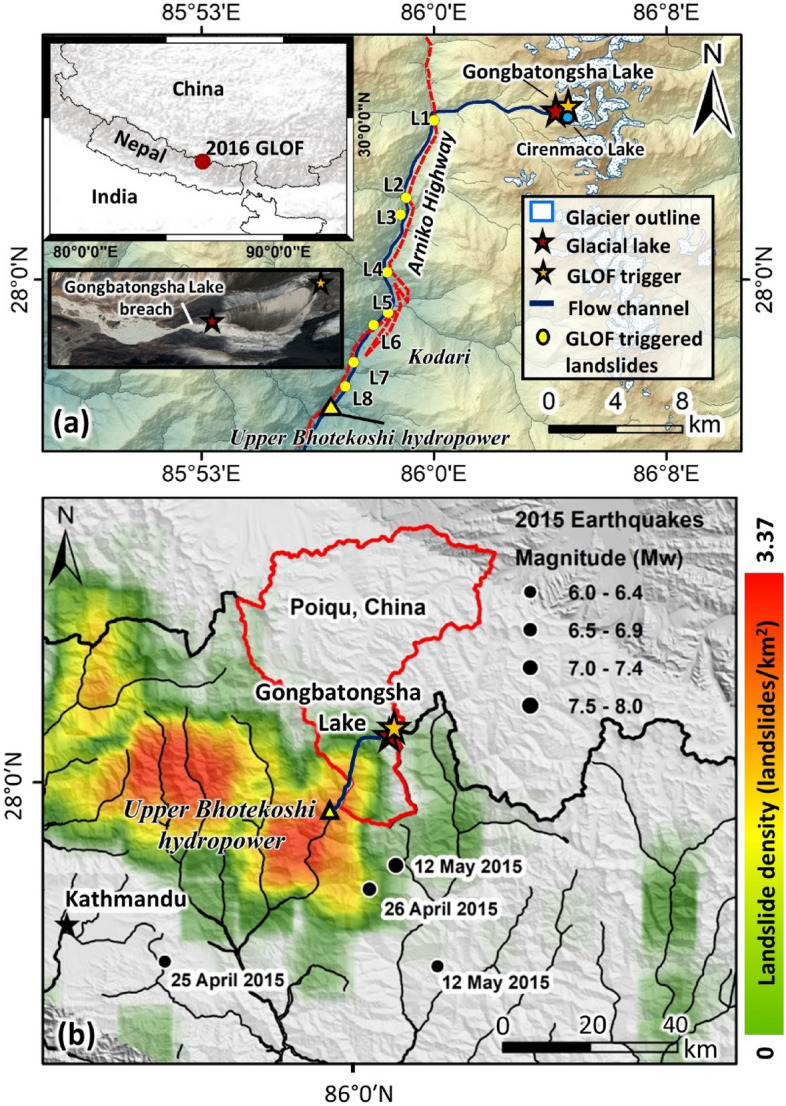
(**a**) Location of the study area showing the Gongbatongsha Lake and the location of the debris flow that triggered the GLOF; marked are the locations of the mapped GLOF induced landslides and the Upper Bhotekoshi hydropower plant; (**b**) Earthquake-induced landslide density map using data from Kagel et al.^29^. Also shown are the (≥ M6) epicenters of major Gorkha earthquake aftershocks. The mainshock was west of this figure. Red outlines the boundary of the Poiqu basin, and blue marks the debris flow channel. This map was generated using ArcMap 10.8 software (© ESRI, https://desktop.arcgis.com). Subset in panel a is from Google Earth (©CNES/Airbus Maxar Technologies).

On 5 July 2016, a debris flow originating in Tibet Autonomous Region at 4610 m elevation crossed the border into Nepal and struck the Upper Bhotekoshi hydropower plant, causing great damage to the plant and various infrastructures, including the Arniko Highway^19,27^ (Fig. 1). This exceptional event originated as a GLOF from the Gongbatongsha glacial lake and later transformed into a severe debris and hyperconcentrated flow, leading to a total economic loss of > 70 million US dollars^28^.

**Figure 2:**
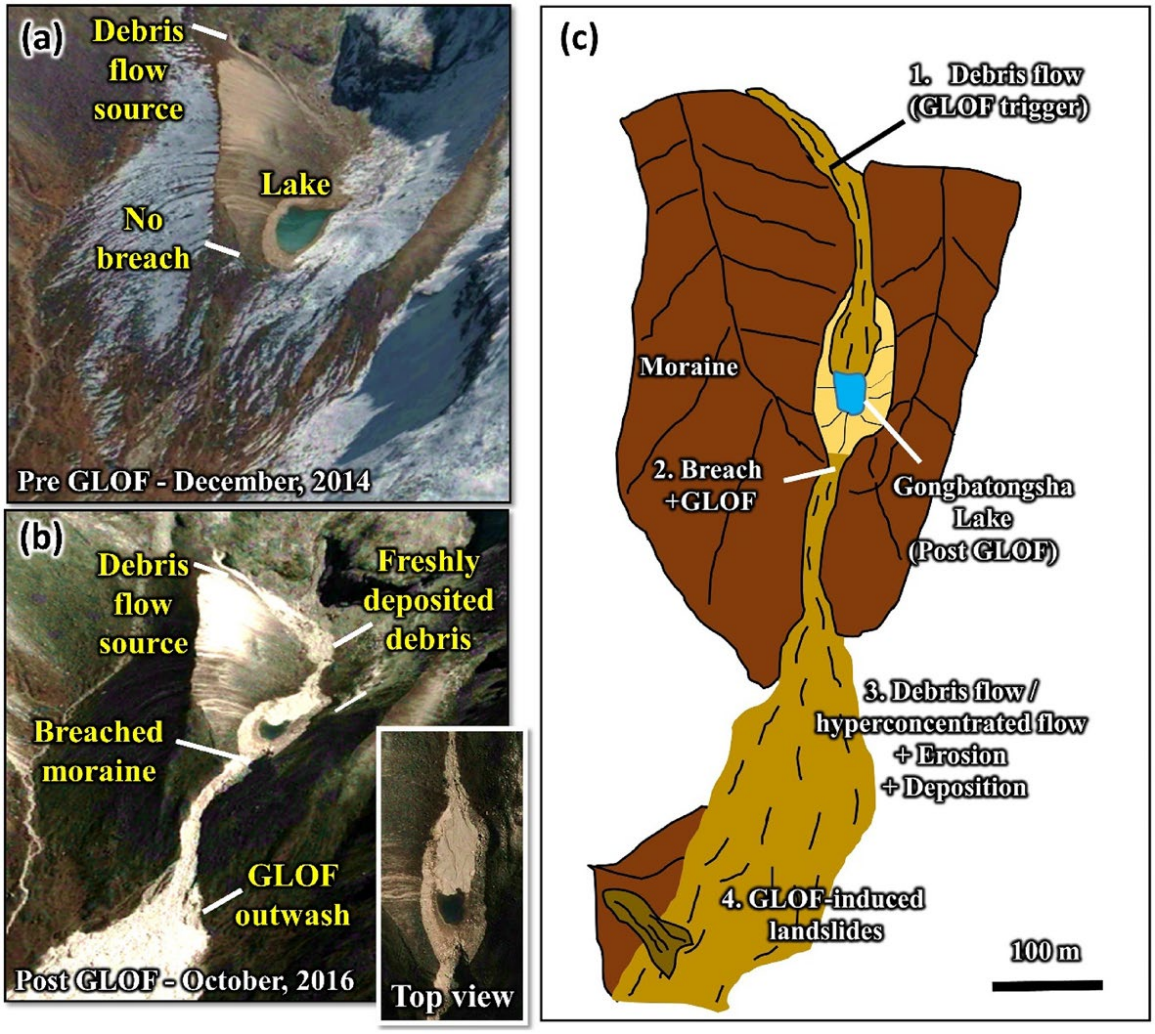
(**a**) Pre-GLOF conditions of the Gongbatongsha Lake; (**b**) post-GLOF imagery showing freshly deposited debris originating from the headwall entering the lake, breached moraine, and GLOF outwash; background imageries in panels a and b are from Google Earth (©CNES/Airbus Maxar Technologies); (**c**) schematic showing the different processes modeled in the study The figure was created using CoralDraw software (URL-https://www.coreldraw.com/en/product/coreldraw).

Unlike Cirenmaco Lake, the Gongbatongsha Lake was small, but it completely drained as fresh debris deposits flowed into the lake from the east slope (Fig. 2), triggering the GLOF that released the entire lake’s volume^19,27,28^ reported to be in the range of 1.1 × 10^5^ m^3^ to 1.7 × 10^5^ m^3^^19,27^. Although the initial volume released from the lake was relatively small, compared to the previous GLOFs in the basin, it transformed into a massive debris flow as it propagated downstream, causing severe geomorphological changes along the valley, and destroying many infrastructures. The effects of this event are comparable to the 1981 GLOF of Cirenmaco Lake, where the flood volume was over 250 times higher than the 2016 Gongbatongsha GLOF (not counting the ingested debris and water from downstream)^25,27^.

The GLOF from the Gongbatongsha lake resulted in a peak discharge of 618 m^3^ s^−1^, but increased to over 4000 m^3^ s^−1^ at the confluence of Zhangzangbo and Poiqu river (http://imde.cas.cn/kydt_2015/201804/t20180404_4991762.html)^19^ as it eroded and entrained the unconsolidated deposits of the channel. The high bedload of the flow even resulted in detectible seismic signals resulting from ground motions induced by the flow^27^. As GLOF propagated downstream, it triggered several landslides, leading to severe damage to highways, bridges, hydropower facilities and caused river blockages. Similar processes were reported in the 1981 Cirenmaco GLOF in the same valley, where the debris flow reactivated several landslide sites and collapsed channel banks^28^.

We believe that the unusual behavior of the Gongbatongsha event requires the ingestion of abundant rock and water mass from along the flow path following the bursting of the lake’s moraine. We reconstructed the different processes to understand the entire event, including how the flow could have attained a much greater volume than the lake’s released flood water. Specifically, the 2016 GLOF included four key stages, (i) debris flow into the Gongbatongsha lake (Tibet Autonomous Region) that originated from the headwall of the lake-facing slope; (ii) lake overfill due to the impact of debris inflow from upstream; (iii) breaching of the frontal moraine, leading to a GLOF; and (iv) transformation of the GLOF into a debris flow and eventually into a hyperconcentrated flow that propagated and caused damages up to 40 km downstream, mainly in Nepal. We present the following hypothesis but save consideration of the acquisition of additional water for the Discussion. Please note, in this work, the modeling focus is on the initial water ingestion from the lake and its flow transitions to debris-flow and hyperconcentrated flow downstream.

There have been limited studies focused on the reconstruction of Himalayan GLOF^8,30^, as it is complex and requires ample data for validation. The present study is a comprehensive reconstructive assessment of the 2016 Gongbatongsha GLOF. Based on the field and satellite-based information, it was reported that the flood had an impact up to ~ 40 km downstream of the lake^27^. However, due to digital elevation model (DEM) artifacts at the Upper Bhotekoshi hydropower dam site located near the China-Nepal border, we restrict our study only up to 23 km downstream of the lake (up to the dam site). Here, we model erosion and deposition due to the GLOF and the flow transformation of the flood to a debris flow and then to a hyperconcentrated flow. We also reconstruct the GLOF-induced landslides along the Zhangzangbo valley and present a numerical model of the debris flow, including flow discharge, transformations, depths, and velocity, and the eroded and deposited volumes along the flow path. The results are validated using published data and high-resolution satellite imagery and DEMs.

### Study region and terrain conditions

The Gongbatongsha Lake (28°04′41″ N; 86°04′12″E) is located in the Zhangzangbo valley, Poiqu/Bhotekoshi basin at an elevation of 4623 m in the central Himalaya. The valley currently hosts seven glacial lakes, of which the Cirenmaco Lake is the largest with a total area of 0.33 km^2^. The Gongbatongsha Lake was a small glacial lake with a total area of 1.7 × 10^4^ m^2^ before the GLOF^27^. The lake is oriented east–west and is surrounded by steep slopes towards the east. The outlet from the lake joins the Poiqu River at Cuoximu, China, after which it flows southwards as Poiqu/Bhotekoshi and crosses the China–Nepal border at Kodari. Abundant debris was available along the river flow path to bulk up the outburst flood (Figs. S2 and S4). This pre-existing debris may have been deposited by co-seismic and post-seismic landslides of the M7.8 Gorkha earthquake that occurred on 25 April 2015 and its largest aftershock on 12 May 2015, the M7.3 Kodari earthquake^29,31^, and by previous GLOFs, landslides, talus, alluvium, and moraines derived from a wide lithologic range of interbedded rock types in the Bhotekoshi valley (Figs. S1, S2, S4, Table S1). It has been reported that the entrainment of these unconsolidated sediments, including Gorkha earthquake- and aftershock-induced landslides (Fig. S2), which in places blocked the main flow channel, caused the amplification of the GLOF^19^, leading to a debris-laden flow downstream^27^. In the supplement, we give the geologic background explaining why landslides are so numerous in the valley and why debris was so abundant and available to the 2016 flow.

## Methods

### Reconstructing debris entering the lake

The analysis of pre- and post-event high-resolution Planet and Google Earth imageries shows that the GLOF must have been triggered by slope failure and detached debris that entered the lake from the lake’s eastern headwall (Fig. 2). To reconstruct this debris flow that impacted the lake, we employed two-dimensional debris flow dynamic module, Rapid Mass Movement Simulation (RAMMS), that was developed to analyze muddy and debris-laden flows in complex terrain^32–34^. RAMMS is a physical-based dynamic model based on Voellmy-Salm finite volume that solves the depth-averaged equations and predicts the slope-parallel velocities and flow heights in two-dimension^35^. The flow characteristics of a mass movement modeled using RAMMS depend on the initial release volume, terrain, and friction parameters given as dry-Coulomb type friction (μ) and viscous-turbulent friction (ξ)^36^. We use the pre-GLOF High Mountain Asia (HMA) DEM (8 m) acquired on 14 November 2015 (HMA_DEM8m_AT_20151114_0506_1040010014461D-00_10400100148CF-E00), where we initially define a release area based on pre- and post-GLOF high-resolution CNES/Airbus imagery from 31 December 2014 and 24 October 2016 showing fresh debris detachment scar originating from ~ 500 m upstream of the lake (see Fig. 2).

It was reported that continuous heavy precipitation in the valley led to slope failure^28^, triggering the GLOF event. For this, we analyzed the time series of the precipitation in the Zhangzangbo valley from June 2016 (Pre-GLOF) to August 2016 using ERA5 total precipitation data (Fig. 4a). The ERA5 data is an atmospheric reanalysis of the global climate that combines model data with observations.

**Figure 3:**
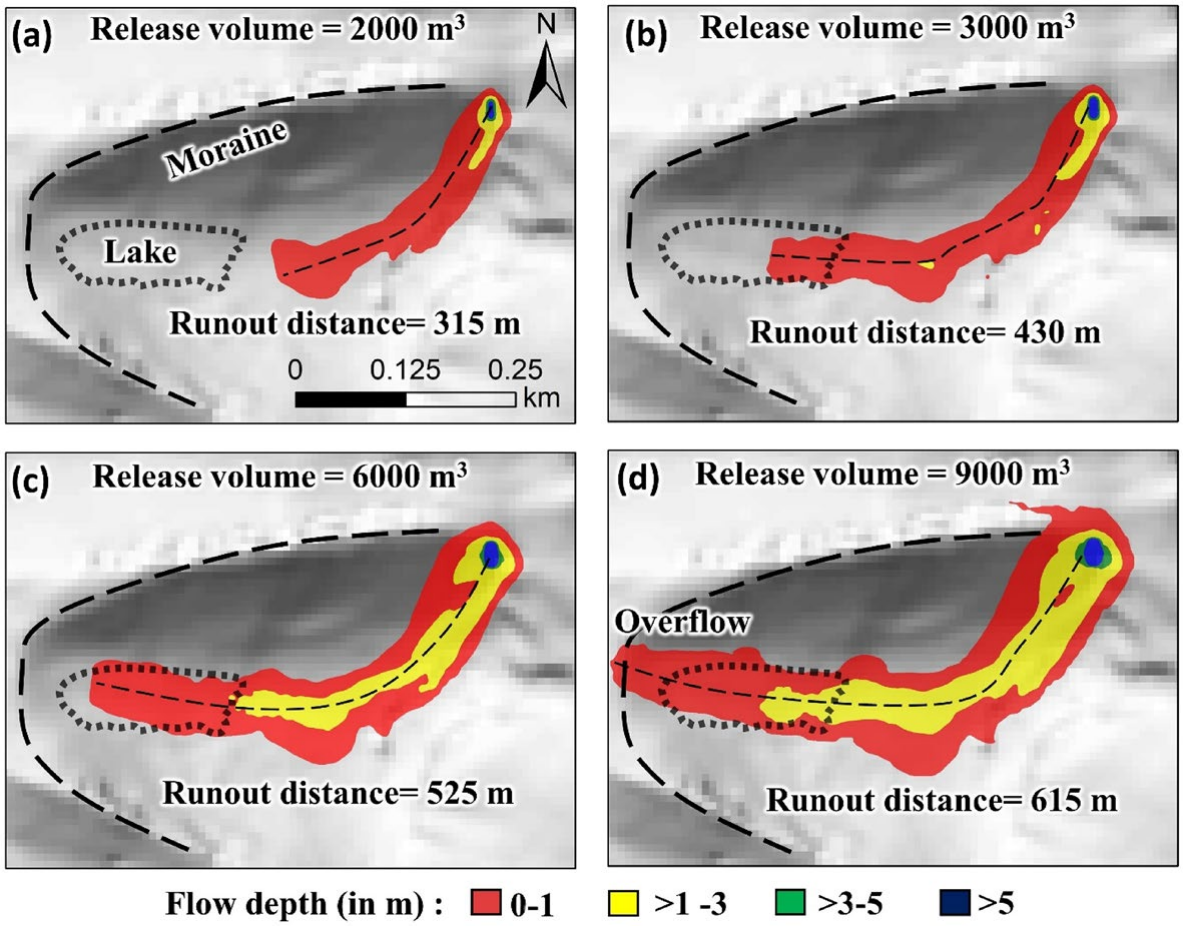
Spatially distributed flow depth (m) and runouts (m) of the detached debris flow that entered the lake for different debris release volumes of (**a**) 2000 m^3^ (**b**) 3000 m^3^ (**c**) 6000 m^3^and (d) 9000 m^3^. This map was created using ArcMap 10.8 software (© ESRI, https://desktop.arcgis.com).

**Figure 4:**
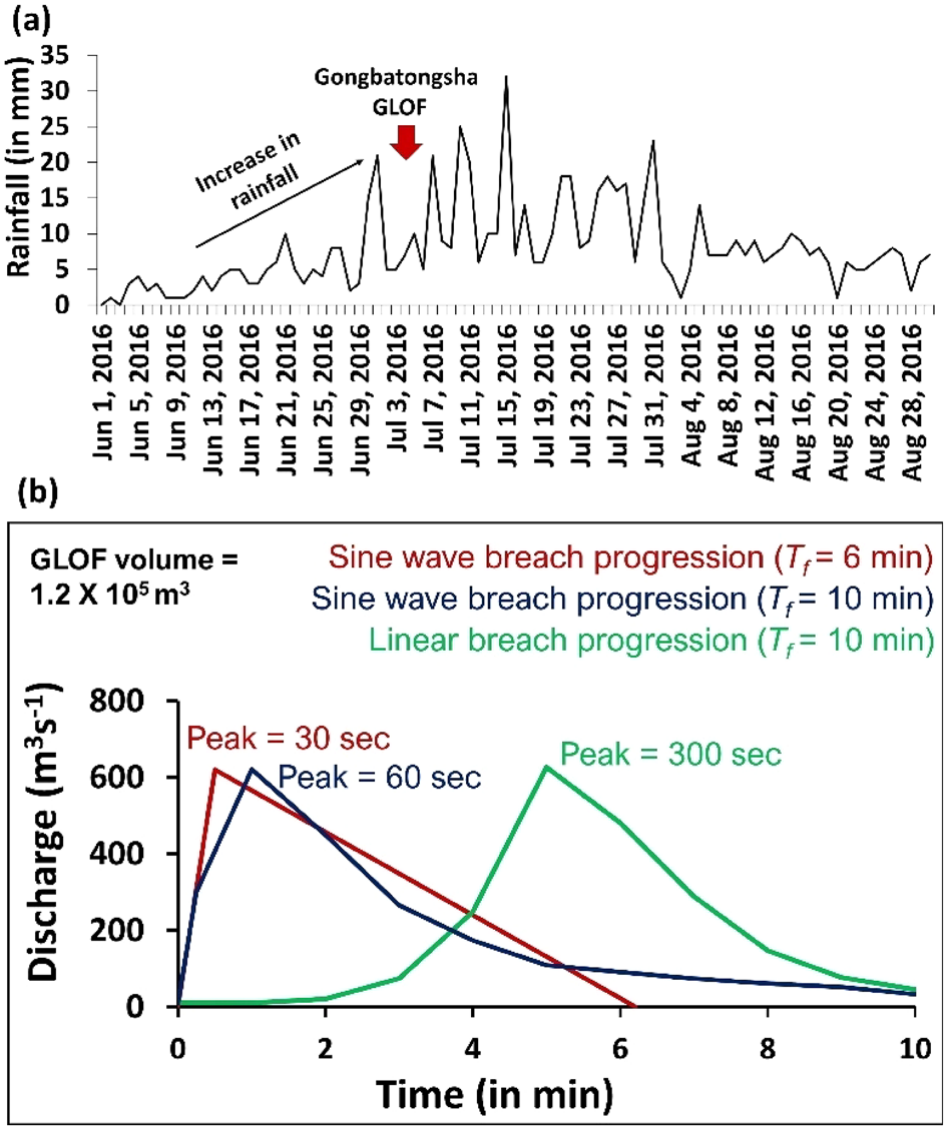
(**a**) Trend of precipitation from June to August 2016; red arrow marks the date of the Gongbatongsha GLOF event; (**b**) Reconstructed GLOF hydrographs of the Gongbatongsha Lake for different lake breach scenarios.

We consider varied release volumes ranging from 2000 to 9000 m^3^ to reconstruct the debris flow that entered the lake (Fig. 3). We rely on the runout distance and debris flow extent to validate our reconstructed flow. Since heavy precipitation existed during/before the GLOF (Fig. 4a), for calibration of the model, we initially consider dry-Coulomb type friction (μ) as 0.2^36^. We changed μ with steps of ± 0.1 until we obtained a flow that best fits the mapped debris-flow extent^36,37^. For viscous-turbulent friction (ξ), we start initially by setting it to 200 m s^−2^. We increase ξ by 100 m s^−2^ to get the best fit. We assume the debris material to be a mixture of ice and rock with a density of 1550 kg m^−2^. The modeled flow that best fit the actual trigger event was used to describe the debris flow characteristics. To interpret the triggering process of the GLOF event that followed debris flow entering the lake, we calculate the total volume deposited into the lake and the impact velocities.

### GLOF reconstruction

We employed the HEC-RAS hydrodynamic model (v 5.0.7) to reconstruct the GLOF hydrograph of the Gongbatongsha lake outburst (https://www.hec.usace.army.mil/software-/hec-ras/)^38–40^. Based on the assessment of the pre- and post-event imagery, it is observed that the GLOF led to the complete drainage of the lake, releasing its entire volume (Fig. 2a,b). Assuming that the debris entering the lake led to overfilling, we model the overtopping failure of the lake. The GLOF event was modeled by performing various moraine-breach simulations such that the entire lake’s volume is drained. The breach width and breach depth were set as 27 m and 9 m, respectively, based on post-event ground observations^19^. We construct three different moraine breach models by varying the breach progression (Linear progression and Sine Wave progression) and the duration it took for the lake to completely drain out (outflow hydrograph duration) in the range of 6 to 10 min. The resulting GLOF outflow hydrographs obtained for different lake emptying durations were compared to the peak discharge of the 2016 event^19^ and are further employed for downstream debris flow modeling.

### Calibration of the debris flow model, reconstructing downstream debris flow, and transformation to hyperconcentrated flow

We first calibrated RAMMS debris flow model input parameters, including upstream boundary condition (GLOF outflow hydrograph), friction parameters (μ and ξ), the density of the flowing material, and erosion parameters based on the available post-GLOF data for validation, and then evaluated various combinations of the above parameters to get the best fit.

The reconstructed GLOF hydrographs were used to model a series of downstream propagation of the flood wave, debris flow, and hyperconcentrated flow using RAMMS. Here, we employ pre-GLOF Landsat imageries, high-resolution google earth imageries, and DEM derivatives to evaluate and map the availability of loose sediments along the flow channel. The presence of loose debris in the Zhangzangbo valley deposited by co-seismic or post-seismic landslides of the Gorkha earthquake on 25 April 2015 is evident prominently in the post-earthquake but pre-GLOF imageries^29,31^. Much of this landslide debris that originated from the river banks and bedrock cliffs was deposited along or adjacent to the flow channel (Fig. S2). We use the earthquake-induced landslides density (log scale) map (Fig. 1b) and DEM of Difference (DoD) to define the erosion parameters in the reconstructive debris flow models. The landslide density map is determined using a neighborhood 1/8° × 1/8° search window (~ 14 km × 12 km) considering the major (≥ M6) epicenters of the Gorkha earthquakes^29^.

Here we initially calculate the post-Gorkha landslide deposition (pixel-based) by evaluating the DoD using HMA DEMs of pre- and post-Gorkha earthquake (see Table S2 for data used). Due to data gaps in the pre- and post-HMA DEMs, the post-Gorkha deposition is only represented in some parts along the channel and is verified with the landslide density map^29^. To reconstruct the pre-GLOF terrain conditions, the calculated deposition (pixels) is added to the pre-GLOF DEM. For the pre-GLOF DEM, we use the Advanced Land Observing Satellite (ALOS)—Phased Array type L-band Synthetic Aperture Radar (PALSAR) DEM acquired on 19 January 2009, with a spatial resolution of 12.5 m (Source DEM 30 m SRTM resampled to 12.5 m). It is to be noted that due to data gaps in the HMA DEM, it could not be considered for hydraulic routing along the channel.

We construct varied debris flow models where we define zones with different degrees of erosion along the flow channel from the lake to the Upper Bhotekoshi Hydropower dam located ~ 23 km downstream of the lake based on pre-GLOF landslide density (Fig. 1b), channel slope, Gorkha landslide deposits (DoD) and other ground reports on pre-GLOF conditions of the valley^19,29^. Here we do not consider channel erosional widening due to model limitations to consider lateral cross-cutting of the valley sidewalls. The first 6.5 km from the lake to the Zhangzangbo-Bhotekoshi confluence, we define zones of viscous debris flow considering relatively high channel slope and available debris. To calibrate the dry-Coulomb type friction (μ), we initially set it to 0.2 and then decreased it by 0.04 until we obtained a flow that is the best fit with the reported discharge at the Zhangzangbo-Bhotekoshi confluence^19,37^. For viscous-turbulent friction (ξ), we start initially by setting it to 200 m s^−2^. We increase ξ by 100 m s^−2^ to get the best fit. The channel takes a sharp bend towards the south at this confluence. Thus, the flow redirects towards the China-Nepal border, where the Upper Bhotekoshi hydropower dam is located approximately 18.5 km downstream from the confluence. Assuming flow transition from debris flow to hyperconcentrated flow, we further tune the friction parameters (μ and ξ). The density of the flow material is set at 1550 kg m^−2^ assuming it to be a mixture of water and debris. The erosion parameters are kept constant along the channel due to high earthquake-induced landslide density and the availability of loose sediments for entrainment (Fig. 1b). However, the final erosion was determined by the flow energy of the GLOF wave. Here, the erosion rate is set at 0.050 m s^−1^, considering the availability of loose sediments along the flow channel. The potential erosion depth (per kPa) is set to 0.200 and the critical shear stress is set to 0.500 kPa^36^.

We calculate debris flow discharge at two cross-sections along the flow channel: one at the Zhangzangbo-Bhotekoshi confluence and the other immediately upstream of the Upper Bhotekoshi hydropower station. The modeled discharge is compared with the Gongbatongsha event^19,28^. We further calculate the total debris volume of the flow along the channel and compare it with the previously reported estimates of available debris along the channel^19^. The modeled flow depths are compared with the mapped trim lines of the debris flow event using post-GLOF Planet Scope imageries. The final reconstructed debris flow is presented where all the above parameters (discharge, flow depths, entrained debris volume) fit best with the actual reported event.

### GLOF-induced landslides

We investigate high-resolution pre- and post-event Google Earth imageries to identify the GLOF-induced landslides in the valley. We map the landslides in the valley within a stretch of 23 km from the Gongbatongsha Lake to the Upper Bhotekoshi hydropower plant. Of the landslides identified, we reconstruct the landslides that either damaged the Arniko highway or blocked the Bhotekoshi river channel. We back-calculate the flow process of these landslides, for which we initially identify and map the source zones and the flow extents. These are further used as proxies to validate the modeled results, for which we used the ALOS PALSAR DEM acquired on 19 January 2009. We present the flow depth and velocities of the reconstructed landslides. We use the RAMMS debris flow module, where the Voellmy friction coefficients are initially set at 0.02 and 200 for μ and ξ, respectively. We increase μ and ξ by 0.01 and 100 m s^−2^ to match individual landslides’ mapped flow and deposition extent. The density of the landslide material is set to 2100 kg m^−2^. We also calculate the total volume of the material deposited in the individually modeled landslides.

## Results

### GLOF trigger and breach outflow

The precipitation trend in the Zhangzangbo valley shows minimal rainfall at the beginning of June 2016 (pre-GLOF), but an increasing trend from mid-June with intense precipitation between June 29 to July 3 (2 days before the GLOF) (Fig. 4a). This was followed by the GLOF on July 5, when 5–10 mm of rainfall was observed. Presuming that continuous rainfall led to a valley headwall/slope failure and detached debris flow into the lake triggered the GLOF, we evaluated the debris flow characteristics. The Voellmy friction coefficient values of 0.2 and 500 m s^−2^ for μ and ξ, respectively, performed well. The modeled debris flow resulted in the runouts of 315 m, 430 m, 525 m, and 615 m for release volumes of 2000 m^3^, 3000 m^3^, 6000 m^3^, and 9000 m^3^, respectively. The flow in the 2000 m^3^ release volume case does not reach the lake (Fig. 3a). The flow in a 9000 m^3^ release volume case overflows the glacial lake basin (Fig. 3d). In this way, the reconstructed volume was determined to be between 3000 and 6000 m^3^ (Fig. 3b,c). The total deposited volume of this debris within the lake is calculated to be 1190–3776 m^3^. The modeled flow velocities range between 13 and 15 m s^−1^ and the lake impact velocities are 2–3 m s^−1^. Here due to the low impact velocity, we interpret that lake overfill to be the likely GLOF trigger that led to overtopping and progressive breaching of the frontal moraine of the Gongbatongsha Lake, following which we constructed a lake breach model, results of which is given below.

GLOF modeling was based on three moraine breach models where the breach width and depth were set as 27 m and 9 m, respectively^19^. The outflow resulted in a total discharge of 1.2 × 10^5^ m^3^ of water. The three modeled GLOFs, one with a lake emptying time of 6 min and two models with a lake emptying time of 10 min, resulted in peaks of 620 m^3^ s^−1^, 623 m^3^ s^−1^, and 621 m^3^ s^−1^_,_ respectively with varying peak times (Fig. 4b). The reconstructed hydrographs are routed downstream considering flow transformations and erosion dynamics. The debris flow hydrographs are compared to the reported debris flow peaks at two cross-sections along the channel. The reconstructed GLOF hydrograph with lake emptying time of 6 m with a Sine Wave breach progression peaking at 30 s validated well with the debris flow discharge downstream of the lake^19,27,28^ (see below for debris flow routing and validation). This emptying time implies an extremely erodible and unstable moraine.

### Flow transformations of the Gongbatongsha GLOF

**Figure 5:**
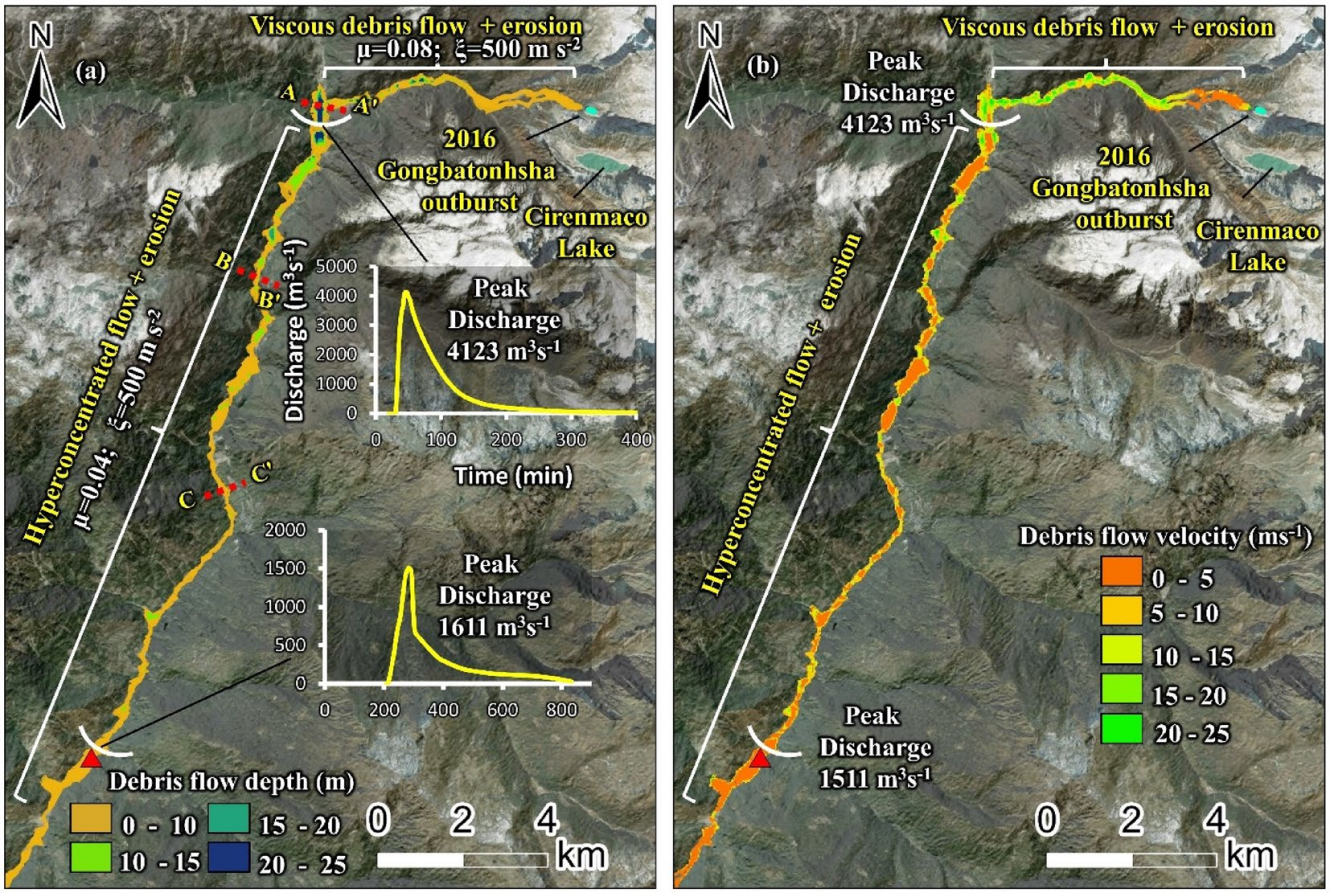
Reconstructed flow hydraulics of the 2016 Gongbatongsha GLOF and flow transformation; (**a**) spatially distributed flow depth (m); (**b**) flow velocity (m s^−1^). Background: ESRI base map overlaid on ALOS PALSAR DEM; (© Esri, Maxar, GeoEye, Earthstar Geographics, CNES/Airbus DS, USDA, USGS, AeroGRID, IGN, and the GIS User Community). This map was created using ArcMap 10.8 software (© ESRI, URL: https://www.esri.com/en-us/arcgis/products/index).

**Figure 6:**
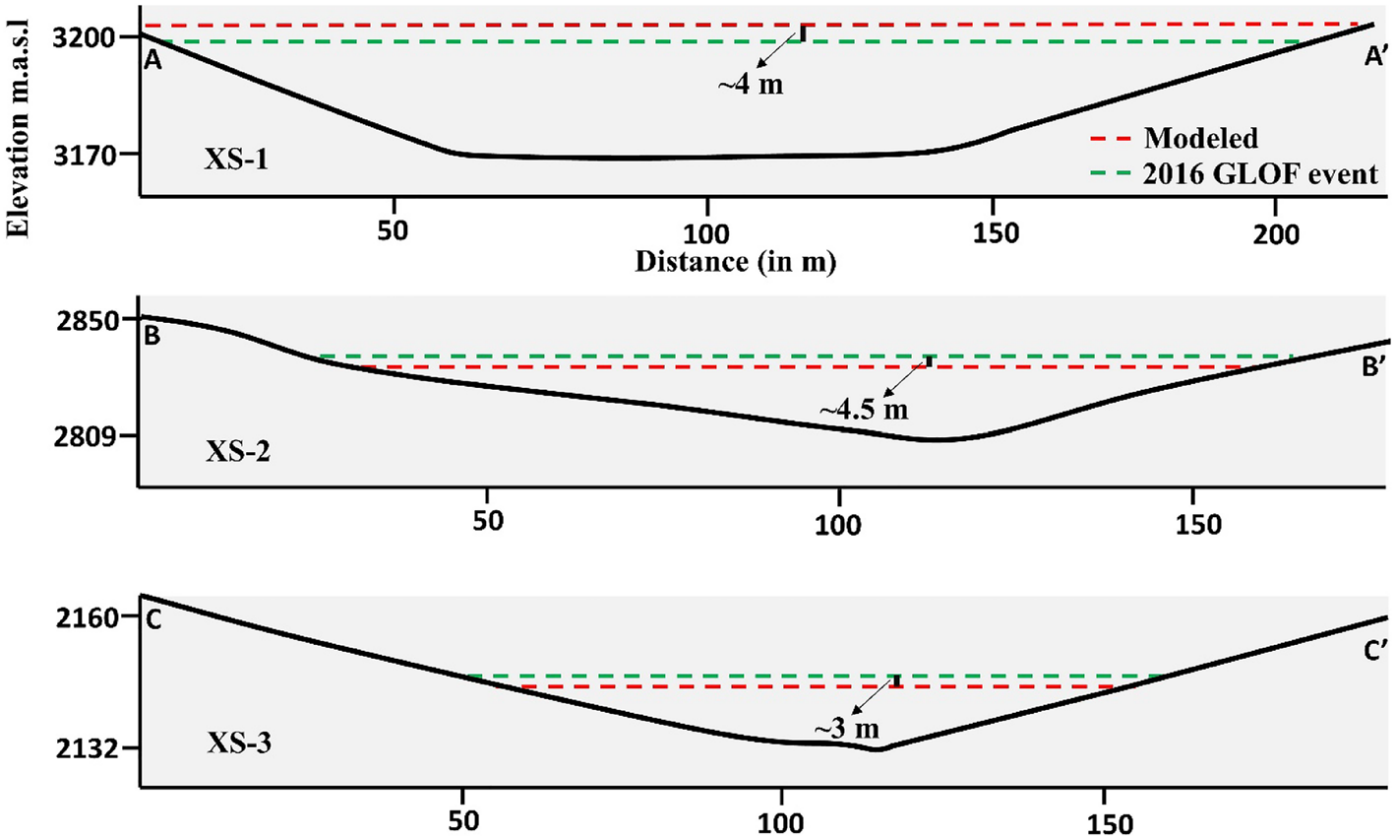
Comparison of the modeled flow inundation and the 2016 Gongbatongsha debris flow along three cross-sections of the flow path; the locations of the cross-sections are marked in Fig. 5a.

Of the three different breach models constructed that resulted in varied outflow hydrographs, we present the results of flow hydraulics that matched with the reported GLOF hydraulics and ground reports, i.e., 6 min of lake emptying and peaking 620 m^3^ s^−1^ at 30 seconds^19,27^. The Voellmy friction coefficient values of 0.08 and 500 m s^−2^ for μ and ξ, respectively, performed well. Based on the erosion rates and the friction parameters defined in the modeling framework, the GLOF transforms into a viscous debris flow, and the peak discharge increases to ~ 4123 m^3^ s^−1^ at the Zhangzangbo-Bhotekoshi confluence (Fig. 5a). This modeled flow discharge aligns with the previously published estimates of 4019 m^3^ s^−1^^19^ and 4000 m^3^ s^−1^^28^. The average channel slope of the section from the lake to the Zhangzangbo-Bhotekoshi confluence is relatively higher than the average slope of the remaining channel up to the Upper Bhotekoshi hydropower dam. The debris flow arrives at the confluence at approximately 20 min and peaks around 47 min after the initiation of the GLOF event. The flow depth at the confluence reaches 25–30 m (Fig. 5). This is mainly due to the sharp bend in the flow channel towards the south, where bulking of the transported debris took place. The modeled flow depths show a good fit with the mapped trim lines based on the post GLOF Planet Scope imageries acquired on 25 August 2016 and high-resolution Google Earth imageries (Fig. 6).

Further downstream from the confluence, where the flow is modeled as a hyperconcentrated flow, the discharge reduced to ~ 1611 m^3^ s^−1^ upstream of the Upper Bhotekoshi hydropower plant located 23 km downstream from the lake. Here the flow depths reach up to 10 m. The mapped and modeled debris flow trim lines are shown along the different cross-sections in Fig. 6. The average modeled velocity of the Gongbatongsha GLOF is calculated to be 5.5 m s^−1^ (Fig. 5b). This is in line with the GLOF velocity calculated as 5 m s^−1^ based on the seismic signals at four different stations downstream of the lake^27^. The debris flow velocity is highest in the first few kilometers until it arrives at the confluence, reaching up to 20–25 m s^−1^. The velocity reduces to ~ 5 m s^−1^ downstream of the confluence (Fig. 5b).

### Erosion and deposition

The total volume of post-earthquake deposits of Gorkha from the lake to the Upper Bhotekoshi hydropower dam calculated based on differencing (DoD) of pre-and post-earthquake DEMs is ~ 3.95 million m^3^. Examples of this type of material are shown in Fig. S2. We recognize that the debris volume can be higher, which could not be determined due to data gaps in the DoD. The deposits show a clear trend when compared to the landslide density map calculated for the major (≥ M6) epicenters (epicenter and locations marked) of the Gorkha earthquake and its aftershocks (Fig. 1b). The DoD deposits are higher up to approximately 3.5 km upstream of the hydropower dam, where the landslide density is highest (~ 2.5 landslides per sq. km) (Fig. 9c,d). The landslide density is reduced upstream towards the lake to approximately 1 to 1.5 landslides per sq. km.

**Figure 7:**
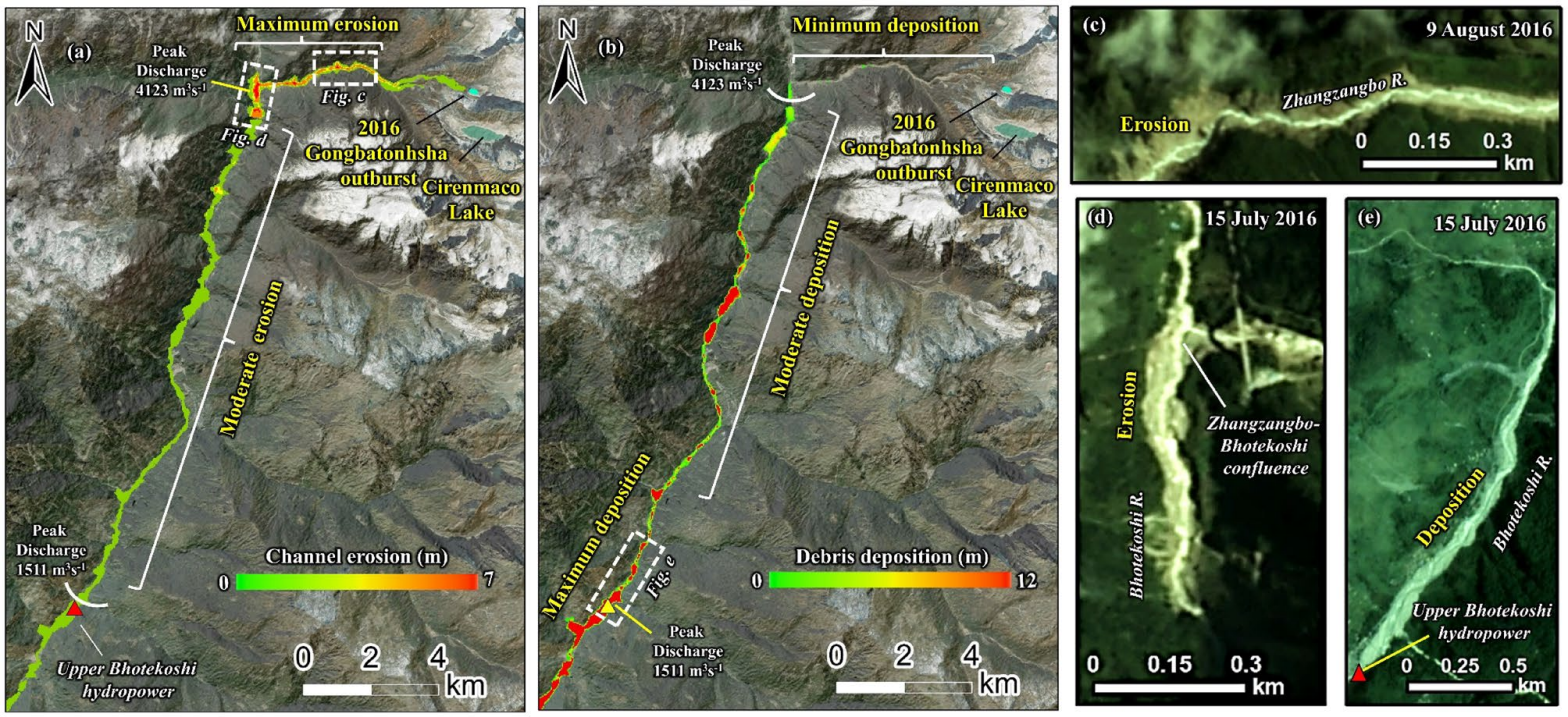
Spatially distributed (**a**) erosion depth (m); (**b**) deposition depths (m) along the flow channel; (**c**–**e**) post GLOF imageries showing erosion and deposition along the flow channel. Background in panels a and b: ESRI base map overlaid on ALOS PALSAR DEM; (© Esri, Maxar, GeoEye, Earthstar Geographics, CNES/Airbus DS, USDA, USGS, AeroGRID, IGN, and the GIS User Community). Background in panels c-e: Planet Scope imageries (©Planet Scope; URL: https://www.planet.com). All maps were created using ArcMap 10.8 software (© ESRI, URL: ).

**Figure 8:**
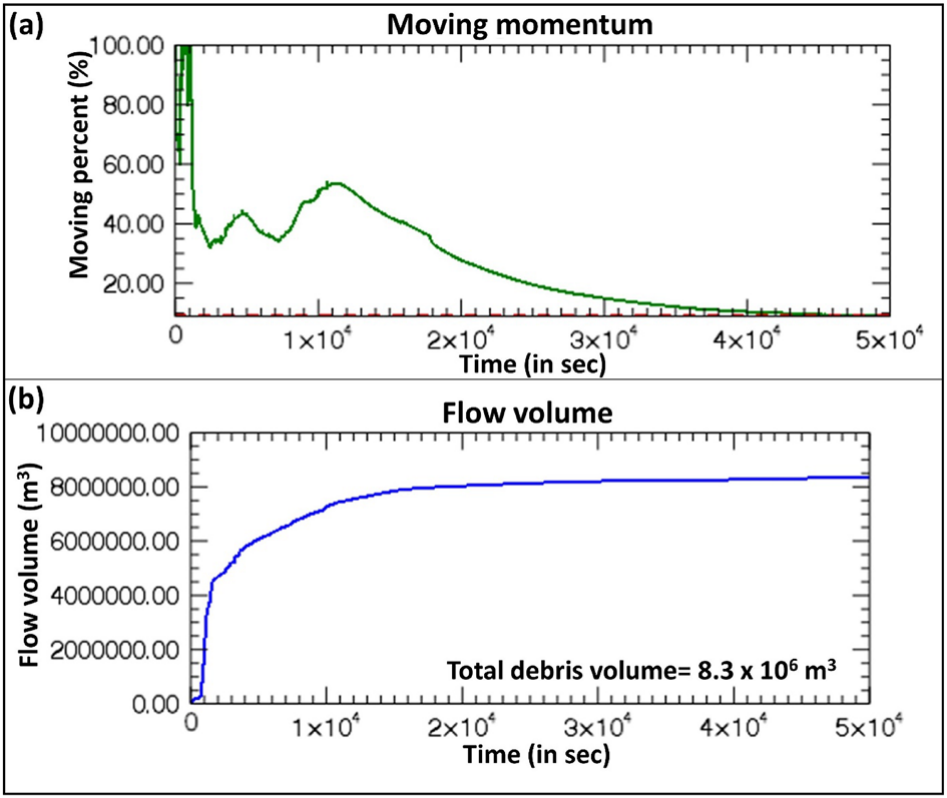
(**a**) Moving momentum (%) of the modeled GLOF/debris flow vs. time (in sec); (**b**) total flow volume (m^3^) vs. time (in sec).

**Figure 9:**
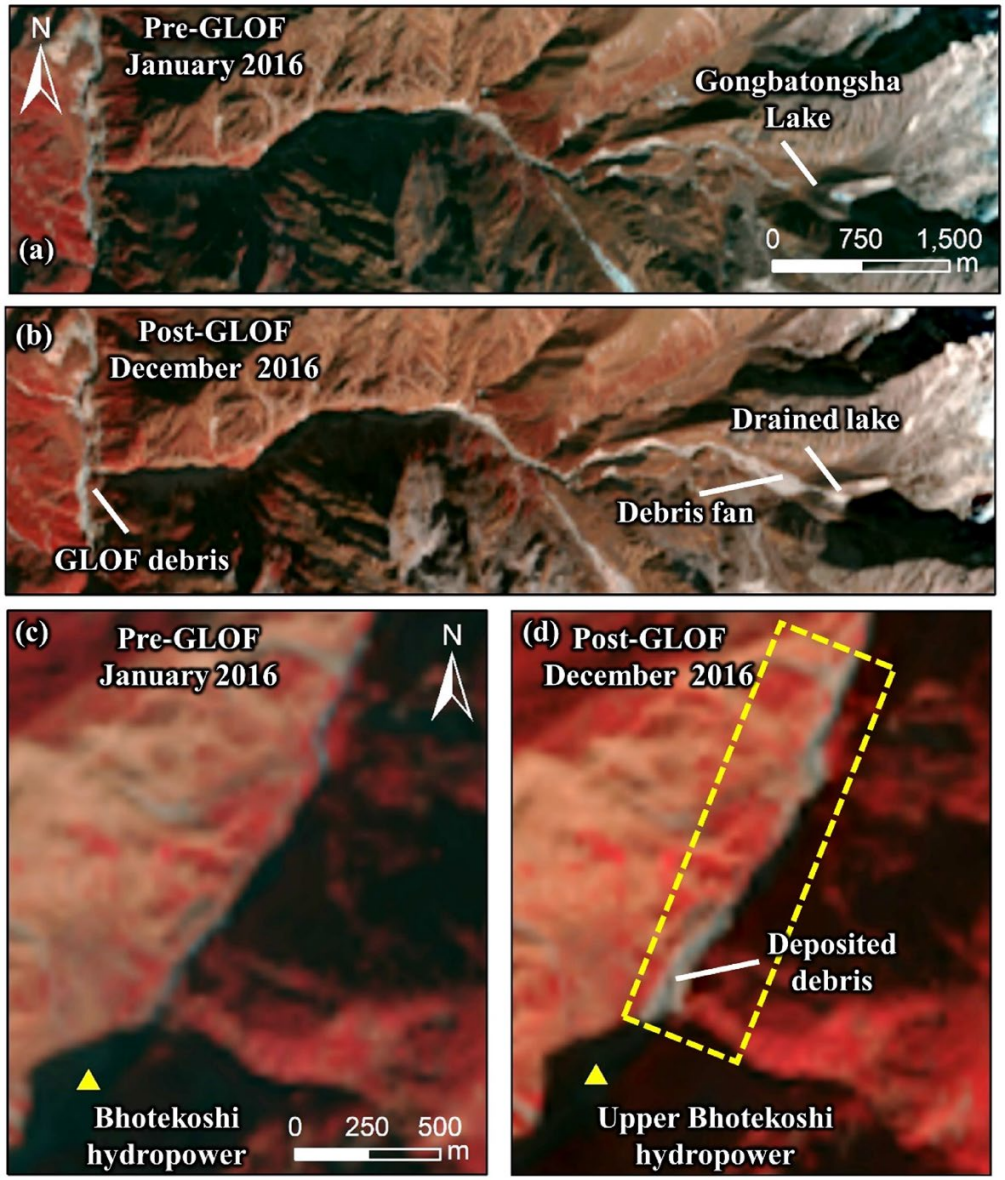
Pre-GLOF Landsat imagery acquired in January 2016 and post-GLOF imagery acquired in December 2016 showing the Gongbatongsha Lake, debris fan, and deposited debris. The satellite images were obtained from USGS Earth Explorer (URL: https://earthexplorer.usgs.gov/). This map was created using ArcMap 10.8 software (© ESRI, URL: https://www.esri.com/en-us/arcgis/products/index).

**Figure 10:**
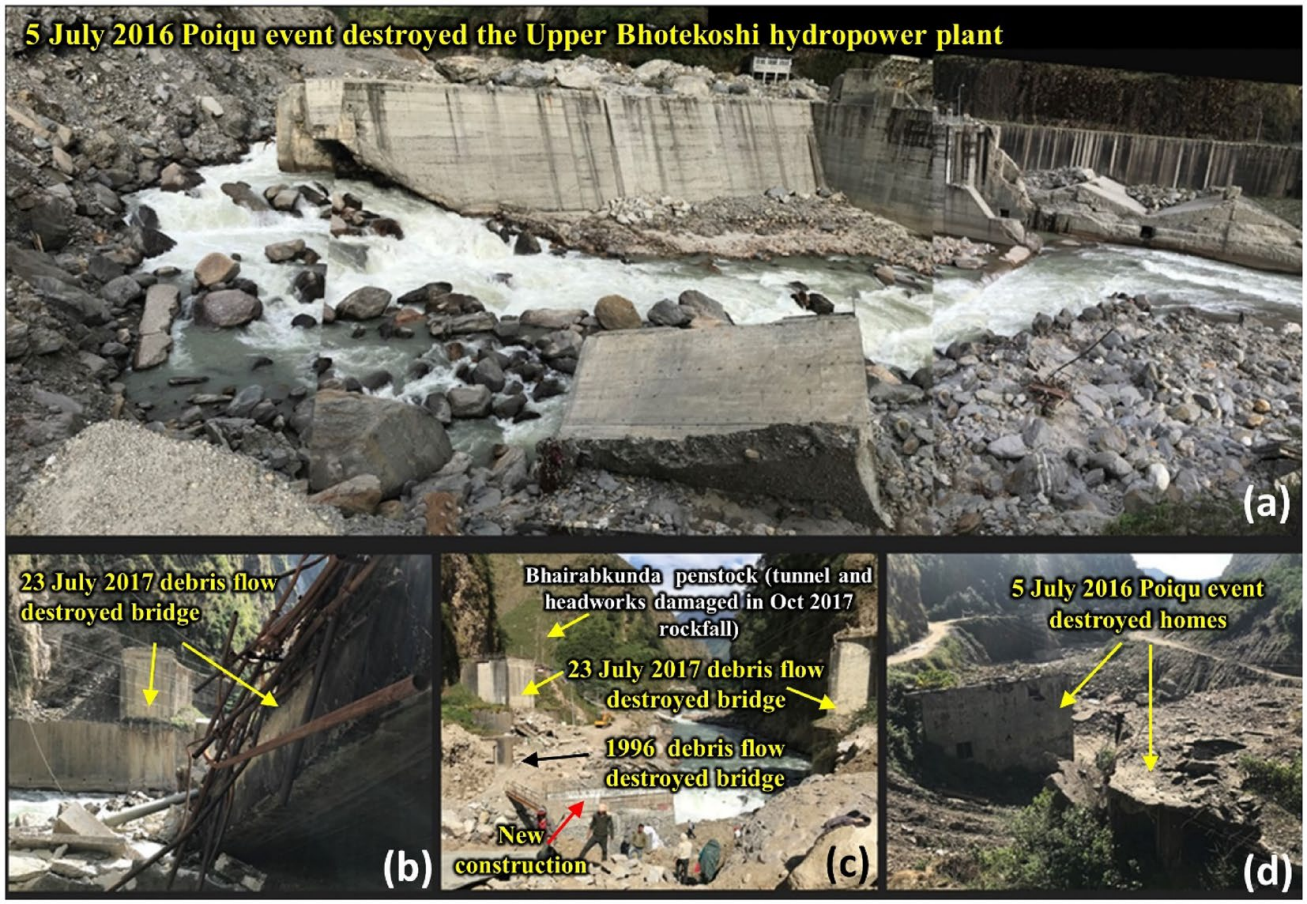
Field photographs of (**a**) the destroyed 45 MW Upper Bhotekoshi hydropower station and nearby infrastructure by the 2016 Gongbatongsha GLOF event (photographed 11/2017). The large boulders (up to 8 m diameter) above and behind the dam were emplaced by the GLOF/debris flow. Power transmission lines were damaged during the 2014 Jure landslide. Multiple key components of the power project were severely damaged during the 2015 Gorkha earthquake^41^ and were being repaired when the Gongbatongsha GLOF struck; (**b**,**c**) The site suffered further impacts from a rockfall in 2017, a fourth consecutive year of disasters. The facility was rebuilt and resumed operations on 22 December 2019. See also Bruen et al.^42^; (**d**) homes destroyed by the 2016 Gongbatongsha GLOF event. The photographs were taken by co-authors Jeffrey S. Kargel and Alina Karki during fieldwork.

The modeled debris flow resulted in maximum erosion of up to 7 m in the stretch from the lake to the immediate downstream of the Zhangzangbo-Bhotekoshi confluence. This is because of the high kinetic energy of the flow that reduces as the flow takes a turn towards the south at the Zhangzangbo-Bhotekoshi confluence (Fig. 7a,d,c). A more detailed map is given in Fig. S3. The flow momentum is the highest during the GLOF outflow and declines rapidly. We interpret that the GLOF transforms into a viscous debris flow, after which the flow momentum starts to decline (Fig. 8a). This can be attributed to the increase in the flow volume as the flow gains eroded debris as it propagates downstream (Fig. 8b). The entrained volume is calculated to be ~ 8.3 × 10^6^ m^3^ (Fig. 8b). It is seen that the deposition process starts at the confluence (Fig. 9a,b) and increases as the flow propagates downstream, while the flow momentum decreases (Figs. 7b and 8b). The maximum deposition is seen to have been occurring near the Upper Bhotekoshi Hydropower dam (Figs. 7e and 9d). A total of 2.1 × 10^5^ m^3^ of debris is deposited within 1 km upstream of the dam site. The field photographs of the damaged hydropower station and large deposited boulders are shown in Fig. 10.

### GLOF triggered landslides

**Figure 11:**
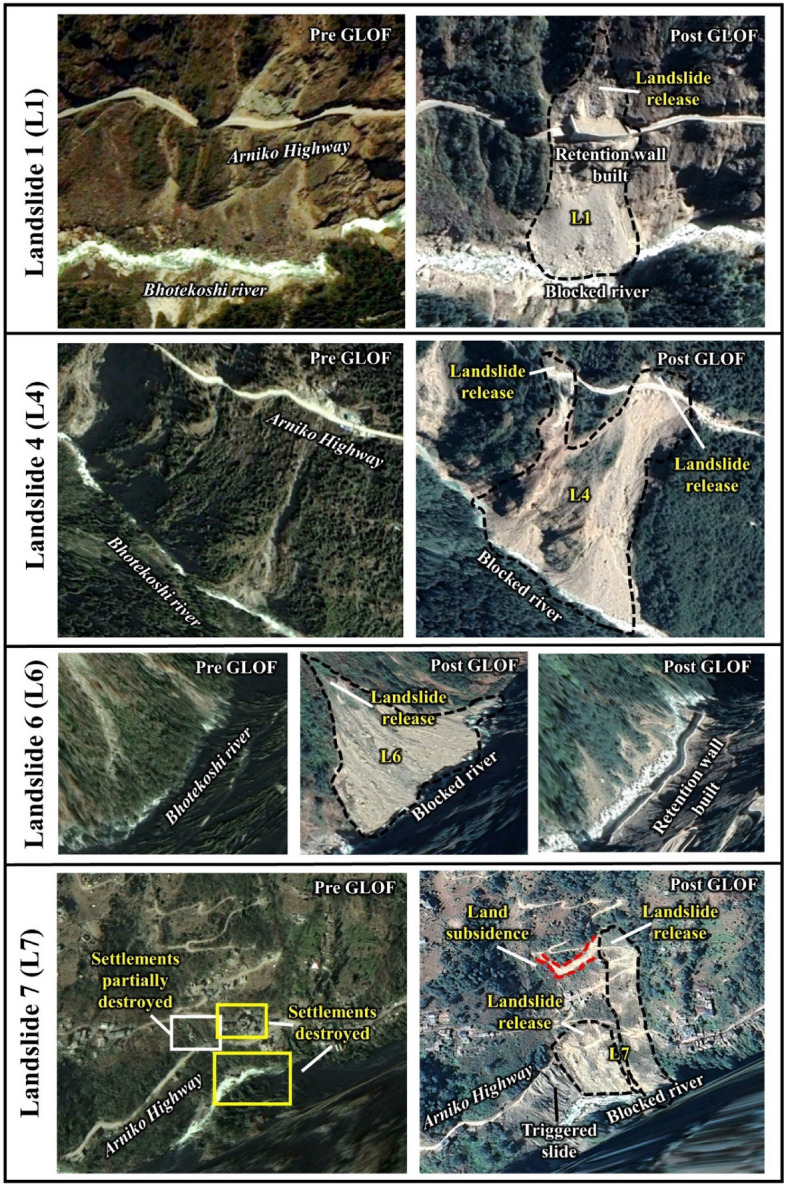
GLOF-induced landslides (L1, L4, L6, and L7) that either damaged the Arniko Highway or blocked the main Bhotekoshi flow channel. Background: Pre- and Post-GLOF imageries from Google Earth (©CNES/Airbus Maxar Technologies).

**Figure 12:**
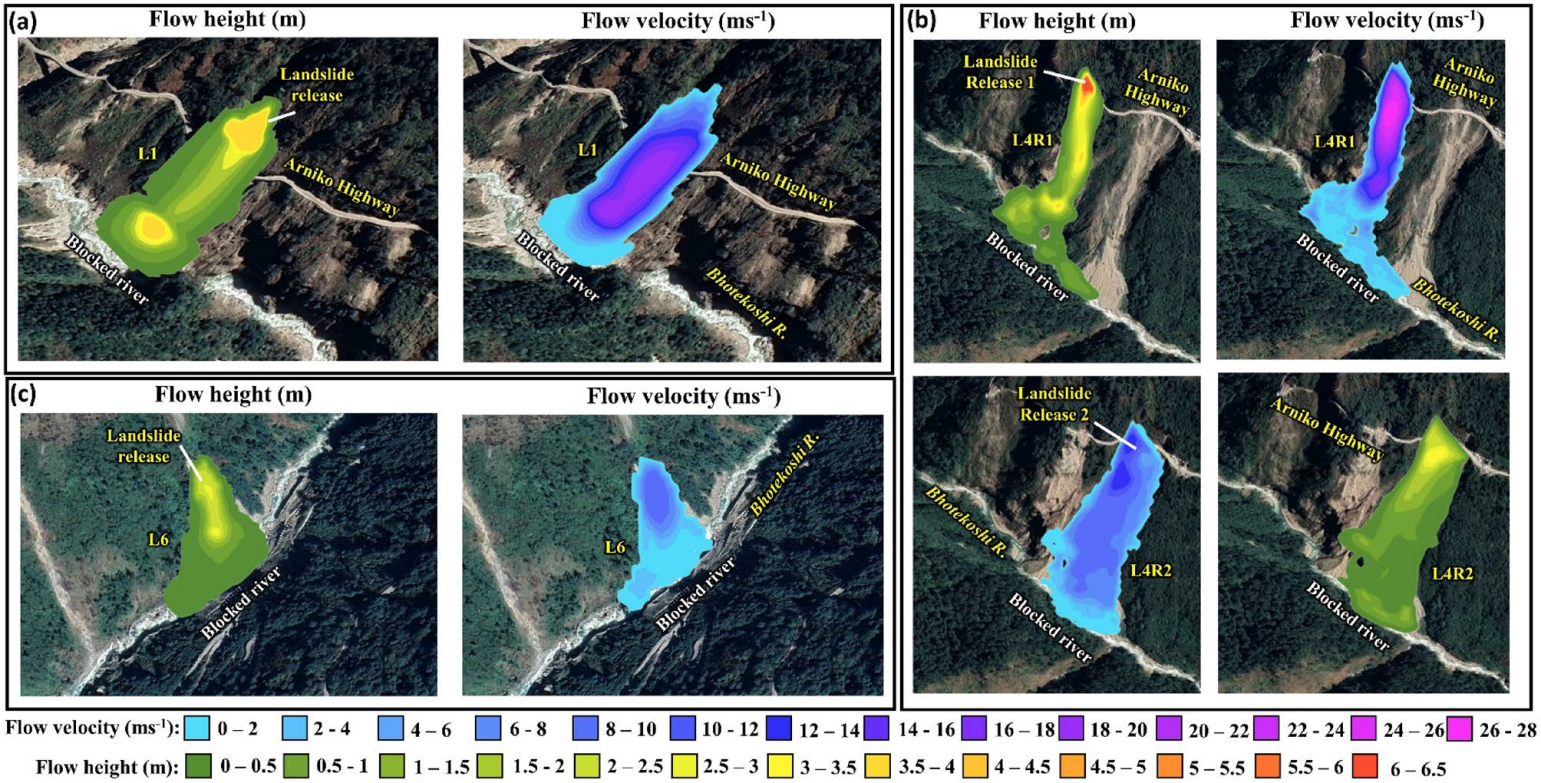
Spatially distributed flow depth (m) and flow velocity (m s^−1^) of the GLOF-induced landslides (**a**) L1; (**b**) L4; and (**c**) L6; Background: imageries from Google Earth (©CNES/Airbus Maxar Technologies).

We mapped eight GLOF triggered landslides (L1 to L8, Fig. 1a; also see Figs. S3 and S5) in the valley from the lake to the Upper Bhotekoshi hydropower plant (see also Fig. S2 for landslides that are believed mainly due to the 2015 earthquakes). Among these eight landslides, four (L1, L4, L6, and L7) either damaged the Arniko Highway or blocked the main Bhotekoshi flow channel (Fig. 11). The release point of L1 was located just above the Arniko Highway and it damaged the highway, following which retention walls were built and a tunnel was constructed for the roadway (Fig. 11). The landslide blocked the Bhotekoshi main channel for 100 m. The flow depth and velocity reach 3–4 m and 15–20 m s^−1^. The reconstructed release volume of L1 is calculated to be ~ 3500 m^3^, of which ~ 3125 m^3^ was deposited on the main river channel (Fig. 12a). Similarly, L4 swept away the Arniko highway at two locations and blocked the main river channel for a 370 m stretch (Figs. 11 and 12b). The deposition depths on the main channel reach up to 2 m. The release zones of the landslide was identified at two locations adjacent to each other from the sidewall of the Arniko Highway (L4 R1 and L4 R2) (Fig. 12b) and the reconstructed release volumes were calculated to be ~ 3000 m^3^ (L4 R1) and 1.3 × 10^6^ m^3^ (L4 R2). The flow velocity reached up to 15–30 m s^−1^ (Fig. 12b). This landslide is one of the largest GLOF-triggered landslides in the valley. The landslide L6 blocked the main channel for 350 m. A retention wall built post-GLOF can be seen along the Bhotekoshi main channel (Figs. 11 and 12c). The reconstructed landslide release volume is calculated to be ~ 4880 m^3^ and the volume deposited on the channel is ~ 4610 m^3^. The reconstructed flow depth and velocity reach 6–10 m and 2–3 m s^−1^, respectively.

### Sources of water

The Gongbatongsha GLOF-initiated debris flow requires additional water beside the lake’s ~ 1.2 × 10^5^ m^3^. There are only two other potential major sources of water: ice and snow mass that may have collapsed into Gongbatongsha Lake along with rock debris, and water already contained in the 18 km stretch of the Poiqu/Bhotekoshi River between the confluence of the Zhangzangbo River and the Upper Bhotekoshi Power Plant. The Bhotekoshi River at the power plant has an annual average flow of 64.6 m^3^ s^−1^ (Bhotekoshi Power Company, Undated)^43^. Considering that a 100-year flood is ~ 695 m^3^ s^−1^, and that the Gongbatongsha GLOF happened during the monsoon, the 18-km stretch of Poiqu/Bhotekoshi River may have averaged ~ 130 m^3^ s^−1^ on the day of the GLOF. If this river stretch’s column-average flow speed was 2 m s^−1^, the river water took ~ 9000 s to traverse that stretch. During that time, the volume of river water was ~ 1.2 million m^3^, which would add to the 120,000 m^3^ of lake water and probably < 50,000 m^3^ that could have been swept up from a 6-km stretch of the Zhangzangbo River. If more water is needed for fluidization, the only other major source would be ice and snow that may have accompanied the initiating rock flow into the lake. We note that the Chamoli disaster in India in 2021 involved a rock: water volume ratio > 4:1 yet was quite fluid^44^. Within plausible values, the Gongbatongsha GLOF-triggered debris flow might have had a comparable rock-to-water ratio, but most of the Gongbatongsha debris flow probably obtained a large and maybe dominant fraction of its water from the Poiqu/Bhotekoshi River.

## Discussions

Outburst floods from glacial lakes are often associated with sediment entrainment and deposition processes^45,46^. These processes can significantly affect the flow behavior, as entrained debris can remain suspended in water and as the debris load increases with the flood propagating downstream, it transforms into a debris flow. These transitional flow behaviors from flood to debris flow or hyperconcentrated flows depends mainly on (i) the kinetic energy (shear stress and stream power) of the flood at a given time and location, which is a function of the slope, discharge, flow velocity, rheology, and volume; (ii) availability and nature of loose debris, for instance, coarse to fine-grained material, e.g., landslide deposits and stream gravels are more easily entrained than large boulders; (iii) geology of the region, e.g., in regions where the bedrock geology is dominated by soft rock, the flooded surface is more susceptible to erosion when compared to hard rock terrains where erosion processes are much slower.

GLOFs around the globe that were associated with debris flows have had severe consequences downstream. For example, the 11 April 2010 GLOF from the Laguna 513 in the Cordillera Blanca transformed into a debris flow event and impacted the city of Carhuaz^37^, debris flow from the 20 April 2017 Langmale GLOF in the Barun valley, Nepal Himalaya, blocked the Arun River^47^; debris flow from the 1941 Palcacocha GLOF in Peruvian Cordillera Blanca claimed thousands of lives and destroyed parts of the city of Huaraz^45,48,49^. The Gongbatongsha GLOF is also a similar event where a GLOF turned into a severe debris flow, causing considerable damage to the downstream areas. The bulking up of loose sediments (most of which were deposited during co- and post-seismic landslides before GLOF) was a major reason for the flow transformation, and that led to an increase in the peak discharge volume of the flood. In the Himalaya, such debris bulking flows were reported previously, e.g., the Dig Tsho GLOF that carried approximately 3.3 million cubic meters of debris and redeposited as the flow propagated downstream^50^. Even though the water volume released in the Dig Tsho GLOF (5.1 × 10^6^ m^3^) was a factor of fifty higher than the volume released in the Gongbatongsha lake (1.2 × 10^5^ m^3^), the transported debris was much higher in the Gongbatongsha GLOF. This hints at the enormous amount of unconsolidated debris already available in the channel that could be easily eroded and ingested into the flow. It is clear from the earthquake-induced landslide density calculated for the major (≥ M6) epicenters of the Gorkha earthquake and its aftershocks (Fig. 1), where very high density is seen along the Gongbatongsha GLOF channel.

The Poiqu basin is a transboundary catchment containing numerous glacial lakes that threaten the neighboring country (Nepal)^16,20^. The basin has a history of past GLOF events that had devastating impacts across the international border of China and Nepal^12,16,20,25,26^. The Gongbatongsha glacial lake was amongst the smallest lakes in the basin and had been categorized as a stable lake^12^. However, this event has highlighted the importance of considering smaller lakes in hazard assessments and mechanisms by which seemingly stable lakes can be destabilized. This same issue pertains to the Langmale event^47^.

Reconstruction of such dynamic events is complex, where erosion and deposition occur at different scales and are spatially variable. Also, as these events occur suddenly and in remote high-altitude regions, the unavailability of ground observations limits the information required for precise reconstructions. Further evaluating the mechanism of flow transformation from the initial triggering of an outburst to floods to debris flows is presently beyond the scope of most numerical models to accurately simulate in detail. However, assumptions to construct a simpler model, as we have done here, may enable a fair reconstruction of such events and to identify the knowledge and modeling gaps that should be filled when it is possible to do so. The present study demonstrated how simple assumptions in modeling a GLOF and its associated processes could help reconstruct such events to the extent that it replicates the original triggering event. The uncertainties arise due to the following: (i) the flow transition remains unknown; here, we assume it to be viscous debris flow in the first part of the flow channel with a relatively steep slope and hyperconcentrated flow in the lower part after the confluence of Zhangzangbo and Bhotekoshi; (ii) the entrained debris during the flow; here we apply a simple erosion model where parametrization of erosion is based on the available entrainable debris along the channel. We use a post-Gorkha HMA DEM where co- and post-seismic landslide deposits are present, but due to data gaps in the HMA DEMs, there are some uncertainties in the total available debris that are challenging to estimate without additional field-based information. We also rely on the pre-GLOF landslide density map to define the model inputs of erosion. We also note that uncertainties persist in reconstructing this GLOF event and are difficult to eliminate because of the unknowns, including flood and debris discharges at different locations along the flow channel. However, the uncertainty in the GLOF hydrograph is expected to be minimum as the breach dimensions were known^19^. For GLOF triggering landslide, the available information to validate our reconstructed flow was runout distance and debris flow extent. Therefore, we give a range of release volume between 3000 and 6000 m^3^. For GLOF outflow volume, we compared it with the empirically calculated (mean-depth based) Potential Flood Volume (PFV) and it had a difference of 17.2%^51^. The lake depth is based on an area–depth relationship obtained from several Himalayan glacial lakes. The transition of the GLOF event to debris flow and further into hyperconcentrated flow is based on assumptions considering the terrain characteristics, including the availability of entrainable debris (pre-GLOF landslide density map) and the slope of the channel. Here we carefully calibrate and tune the friction parameters µ and ξ and use proxies to validate our results, including discharge, inundation, and flow velocities. We also confirmed this with field reconnaissance conducted after the GLOF and the large size of debris that were deposited around the Bhotekoshi hydropower plant. Comparing the modeled flow inundation and the 2016 Gongbatongsha debris flow along three cross-sections resulted in an RMSE of 3.8 m. We assume it to be a cumulative uncertainty of DEM and the model parameters, as constraining them individually is challenging. We back-calculated the flow process of the GLOF-triggered landslides where the tuning of µ and ξ was applied to validate the model results. However, uncertainties in the release volumes could not be avoided due to the unavailability of ground data. We note that the uncertainty can be further constrained provided more ground data is available for validation.

The Poiqu basin has a GLOF return period of 30 years^27^, which means continuous monitoring is needed. The basin had an EWS with five sensors and an automatic siren system installed after GLOF from the Cirenmaco lake, but the EWS collapsed during the Gorkha quakes in 2015 and is no longer functional. Therefore, no warning was issued during the Gongbatongsha GLOF, and the event led to a loss of about USD 66.35 to 77.34 million. The Department of Hydrology and Meteorology (DHM) then installed a flood sensor near the Friendship bridge after the Gongbatongsha GLOF. The Poiqu basin has a long history of glacier-related floods; therefore, there is a great need to monitor potential occurrence of these events, identify precursor hazards, and improve the EWS in the transboundary region between China and Nepal. Bilateral efforts on reducing GLOF risk are in great need for this region.

## Conclusions and future work

We reconstruct the 2016 Gongbatongsha GLOF in the Poiqu basin, in the central Himalaya. The proxies of flow discharge, breach parameters, and Gorkha landslide deposits are successfully employed for the debris flow model implementation and reconstruct a complex process in the event involving debris entrainment and flow transitions. The Gongbatongsha GLOF was an exceptional event in the Poiqu basin where the severity of such a small lake outburst was unexpected. This GLOF is a classic example where debris entrainment intensifies the flood into a debris-rich flow, causing great destruction downstream. Our study highlights the processes of the Gongbatongsha debris flow, including its onset, propagation, flow transformations, erosion, and deposition. Despite some limitations of the debris flow model (RAMMS) or other similar computational models, the event was usefully reconstructed. Modeling results show that the significant increase in peak discharge from 618 m^3^ s^−1^ to 4123 m^3^ s^−1^ at 6.5 km downstream from the lake resulted due to the erodible nature of the terrain and sediment bulking by eroded sediments at the Zhangzangbo-Bhotekoshi confluence. The peak discharge immediately upstream of the Upper Bhotekoshi hydropower was calculated to be 1611 m^3^ s^−1^. The total debris entrained in the event was calculated to be ~ 8.3 × 10^6^ m^3^. The modeling indicates that an initializing landslide of a few thousand cubic meters progressively amplified by three orders of magnitude first by release of glacial lake water and then ingestion of down-valley sediment and river water to create a huge debris flow. More field investigation of the terrain and sophisticated models can be helpful to understand the event in further detail. Advanced numerical modeling capabilities are needed to capture some of the phenomenologies of such complex events and empower disaster risk reduction in vulnerable areas. Regular monitoring of the glacial lakes, including the smaller lakes in the basin, is recommended. Further, most of the lakes in the Poiqu basin pose transboundary threats; hence, the methodology presented in this study can be used to generate scenario-based models for these lakes, which will help policy/decision-makers understand hazards complexity in the basin. It may also allow them to improve the EWS network in the region to avoid and reduce the impact of future catastrophes.

## Supplementary Information

Supplementary Information.
