# Supplementary material for: Transition of a small Himalayan glacier lake outburst flood to a giant transborder flood and debris flow
Source: Sci Rep. 2022 Jul 20;12:12421. doi: 10.1038/s41598-022-16337-6 (PMC9300610; doi:10.1038/s41598-022-16337-6)
Supplement: Supplementary file 1 — Supplementary Information. [file 41598_2022_16337_MOESM1_ESM.docx]

**Supplementary Materials**

**Transition of a small Himalayan glacier lake outburst flood to a giant transborder flood and debris flow**

**Ashim Sattar*^1,2^, Umesh K. Haritashya^1^, Jeffrey S. Kargel^3^, Alina Karki^4^**

**Correspondance-ashim.sattar@gmail.com**

^1^ Department of Geology and Environmental Geosciences, University of Dayton, Dayton, OH 45469, USA

^2^ Environment and Climate: Impacts, Risks and Adaptation (EClim), Department of Geography, University of Zurich, Switzerland

^3^Planetary Science Institute, Tucson, AZ 85719, USA

^4^Nepal Electricity Authority, Kathmandu, Nepal

*Correspondence to: [ashim.sattar@gmail.com](mailto:ashim.sattar@gmail.com)

**S1: Bedrock lithological variability in the Himalaya is a critical but understudied hazard factor**

Bedrock lithological variability is a recognized important but understudied factor in the behaviors of destructive landslides, floods and debris flows^1-5^. Distinct lithologies along bedding planes, joints, and faults control groundwater flow. Hydrolysis, formation of phyllosilicates, dissolution, and water saturation weakens rocks along those planes and lowers the coefficient of static friction, producing potential bedrock slip planes and high susceptibilities to wedge failures. Those structures also juxtapose different rock units at the surface, where differential rates of hydrolysis, dissolution, frost shattering, and stream erosion and seeps undermine outcrop overhangs. Some interbedded rock types are intrinsically weak, thus adding to a potential for slope failures. Lithology also controls debris production rates by fluvial, glacial, and freeze-thaw processes. Furthermore, bedrock lithology controls the properties of soils, e.g., particle grain size, porosity, permeability, water saturation, and geotechnical properties pertinent to creep and erosion. Thus, sedimentological and tectonic structural juxtapositions of differing lithologies affect the surface relief and internal friction properties of rock and can affect the sudden failures of bedrock wedges, collapses of overhangs, sudden slips of ice or soils over bedrock, and soil creep and flow. These failures can be triggered by seismicity (as in the 2015 Gorkha earthquake) or extreme weather, such as protracted rainfall or exceptional snowmelt.

Geological factors controlled the generation of debris and the development of soil geotechnical properties in the Poiqu-Bhotekoshi Valley. The 2016 flood accessed and ingested this debris. It is not known how the specific properties of the valley’s debris accumulations relate to its ingestion and the evolving rheology and behavior of the flow.

Considering these geological factors, we provide supplemental information on the Poiqu-Bhotekoshi basin’s bedrock geology. The 2016 GLOF had its source in the Higher Himalaya Crystalline units. As the flow passed into Nepal and the map area of Figure S1, the lithologies in the valley are dominated by the Lesser Himalaya’s interbedded sedimentary and metasedimentary rocks (Fig. S1 and Table S1). The Nepal Department of Mines and Geology (1984) mapped the rocks as the Lakharpata and Dailekh subgroups. These units contain diverse rock types, with bedding and other structures occurring on a wide range of scales, from thin lamellae to massive beds— a feature that contributes to high susceptibility to landslide, erosion, and debris production (Karki and Kargel 2017). Mapping by different groups has differed in the delineation of formations, nomenclature, and geochronology, especially across different regions of Nepal, but the lithological identifications are similar (Table S1). The valley also is riddled with joints and faults (Figures S1, S2F, and G, S4C). Many of these structures are involved in channeling groundwater and surface water flow (Figure S2G), where bedrock weathering and geomechanical weakening are accentuated.

Weak beds in the Bhotekoshi Valley, such as of shale, phyllite, highly micaceous or graphitic schist, and even gneiss if it is heavily weathered, can readily fail. Massive, strong overhanging beds, such as of quarzite, can then ride along in landslides. Groundwater-saturated zones in interbedded strata, especially where seeps have undermined cliff faces, are particularly susceptible to landslide failures, as apparently was the case for the huge 2014 Jure landslide (location in Fig. S1) just down the Bhotekoshi Valley from the area that was most affected by the 2016 GLOF. Decades and centuries of landslides, erosion and fluvial transport have deposited substantial sediment in the Bhotekoshi Valley. The 2015 Gorkha earthquakes added to the debris in the areas affected by the 2016 flood (Figures S2, S3, S4). The bedded sedimentary and meta-sedimentary rocks are thus a key basis for how the 2016 event bulked up in volume and explain why so many secondary landslides were triggered by the GLOF and debris flow (Figure 11). These processes were important in 2016’s rheological and behavioral evolution and the destruction it caused downstream (Figures 10, S4, S5). The details remain to be investigated.

Several major thrust faults have tectonically disturbed the rocks in the border area shown in Figure S1 and have juxtaposed distinct lithologies, leading to high risks of destructive landslide failures and abundant debris production. For example, repeat satellite imagery, the area of Kodari and Larcha, right on the border, show almost yearly reactivation of landslides by the summer monsoon and also the 2015 Gorkha earthquake, and both the precipitation event and Gonbatongsha GLOF of 2016. The repeat destruction of the Larcha Bridge in 1996 and 2017 (Fig. S4A, B), for instance, is due to reactivated landslide activity sourced in the area of the thrust faults marked in Figure S1. Likewise, the 2016 GLOF reactivated landslides that were active just the year before due to the Gorkha earthquake. The 2014 Jure landslide was a massive reactivation of landslides from immediately preceding years. And so it must be assumed that many more such major reactivations will continue, and the disasters will increase in frequency and magnitude as the valley is developed, reflecting a point made many times recently that so-called natural disasters are actually man-made.

Such geologic controls on debris production are recognized in the scientific literature, but they are not typically considered in detail for landslide, debris flow, and glacial lake outburst flood hazard assessments. The 2016 Gongbatongsha GLOF is particularly remarkable for having ingested so much debris and water that it amplified its magnitude by three orders of magnitude from the initiating rock failure. Since landslides commonly develop on individual bedding planes or along individual tectonic discontinuities, the understanding of landslide and debris flow hazards ought to be built on an understanding of the bedding-scale and fault-scale structure, rather than generalized geologic mapping. This may be considered a major gap area for training, research, and applications in Himalaya. Landslide, debris flow, GLOF hazard, risk, and disaster studies should include detailed geologic and bed lithology and surficial debris mapping, geomechanical testing, and groundwater studies.


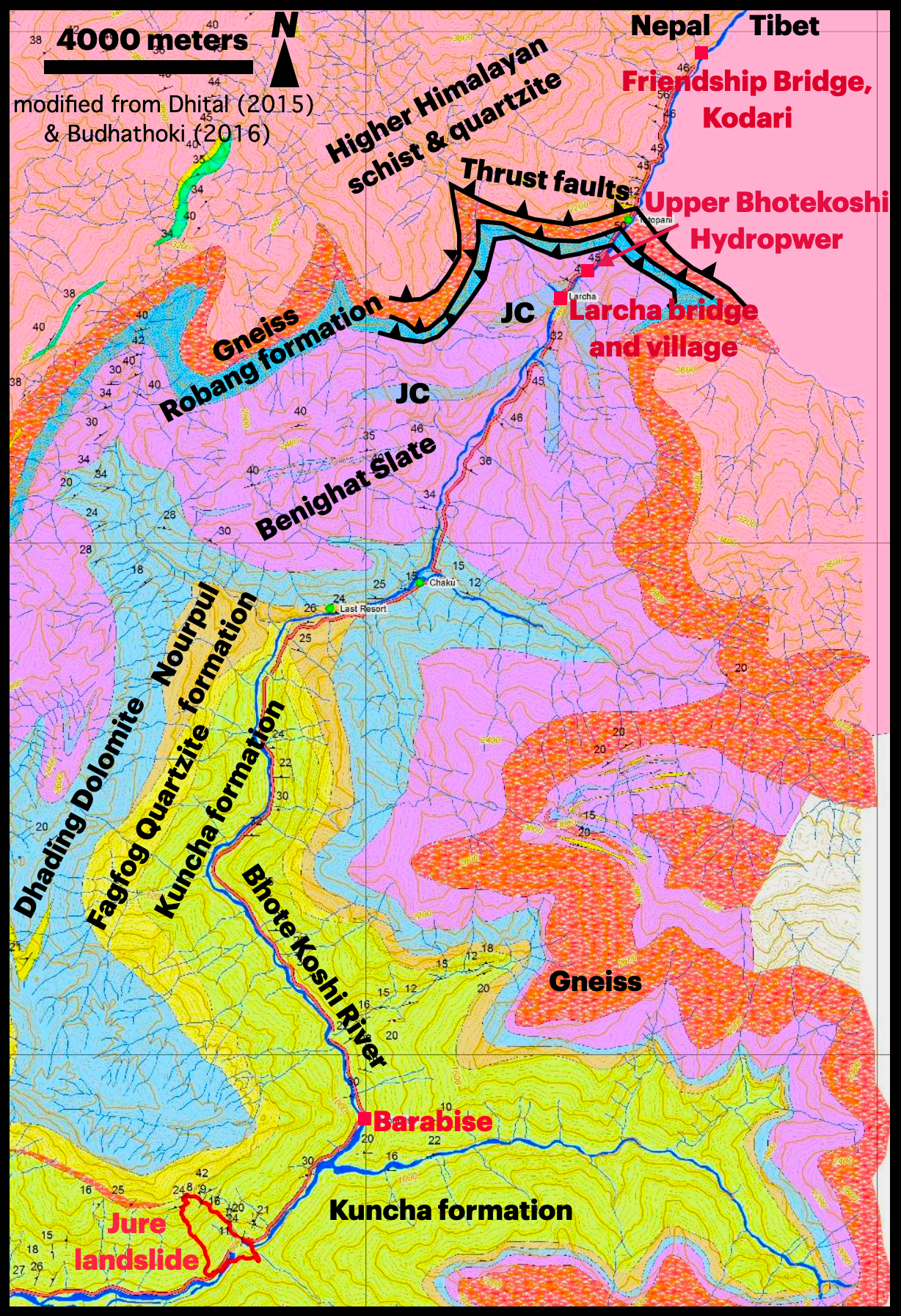


**Fig. S1.** Geological map of the Bhotekoshi river area, modified from Dhital (2015)^6^ and Rajendra Budhathoki (2016)^3^; JC is the Jiku carbonates. This map was modified using ArcMap 10.8 software (© ESRI, https://desktop.arcgis.com).

**Table S1.** Lithologies in the Lesser Himalayan region of the Bhotekoshi Valley according to two correlated stratigraphies.


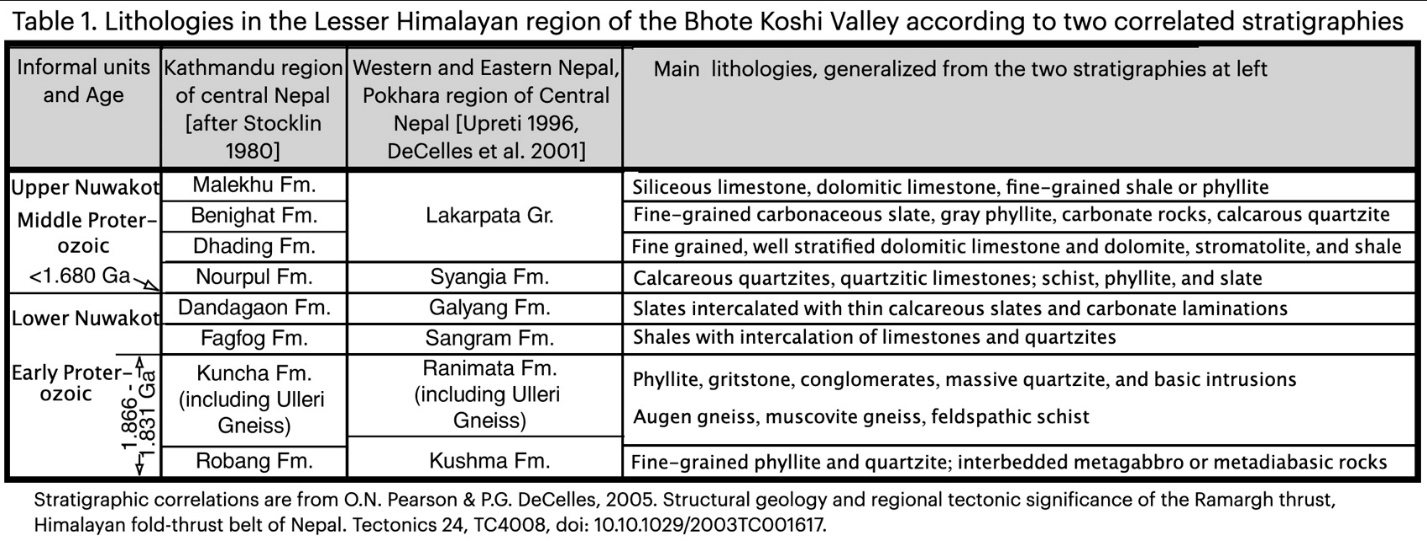


**
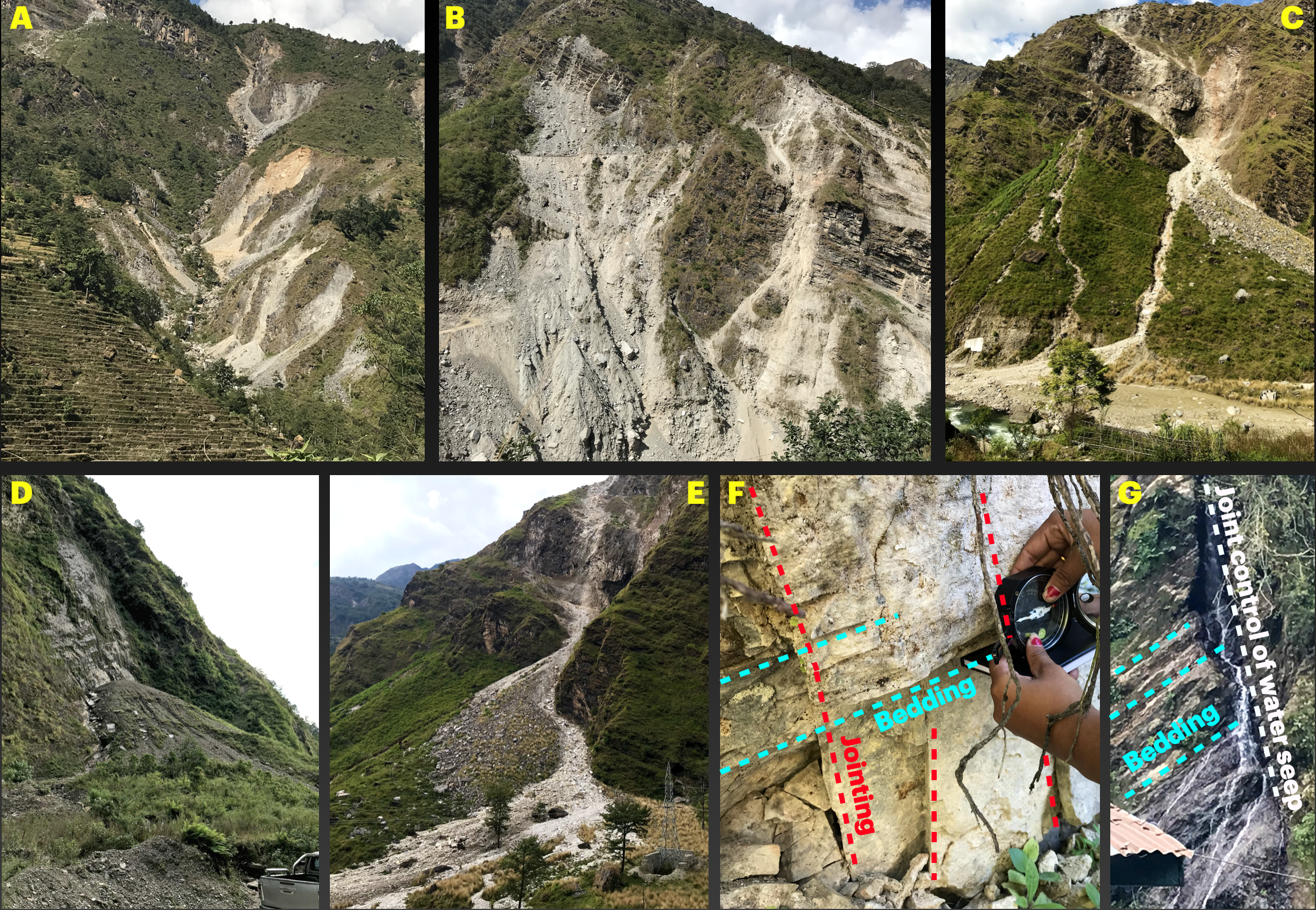
**

**Figure S2.** Recent landslides sourced in Lesser Himalaya in the Bhotokoshi Valley in the area affected by the 2016 event (A-E). Some debris was available for pick-up by the 2016 GLOF. (F) Friable, geomechanically weak, and heavily jointed shaly phyllite near a large Chinese-funded hydropower project that is under construction in the area that was affected by the 2016 GLOF. (G) Example of joint-controlled water seepage and runoff. Photos by J. Kargel, Nov. 2017.


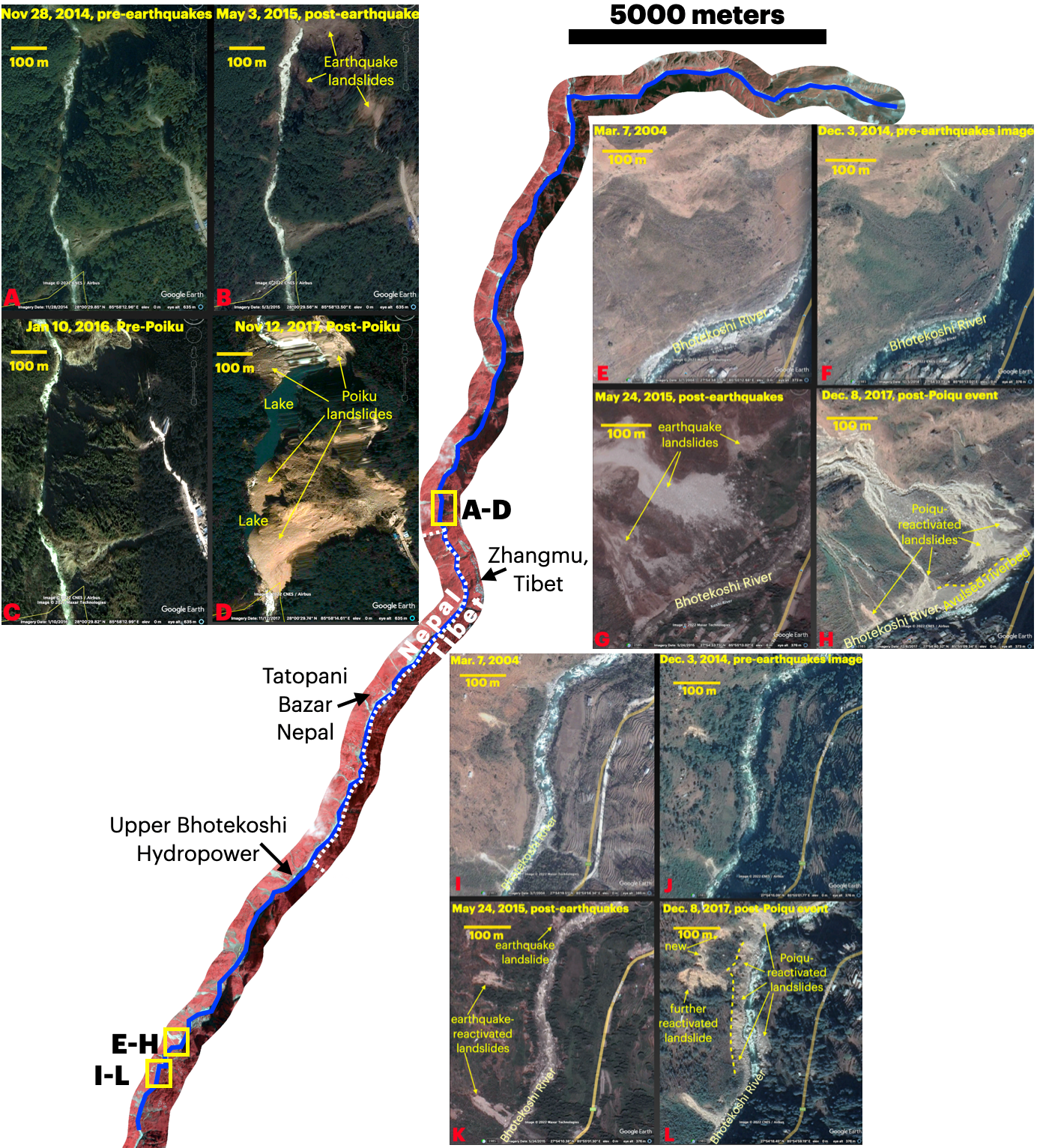


**Figure S3.** Time series (images from Google Earth, ©CNES/Airbus, Maxar Technologies) development of landslides along the Bhotekoshi/Poiqu River. The False Colour Composite (FCC) ‘strip’ is a cropped mosaic of Planet Scope images (3 m/pixel) acquired on Sep. 30, 2016 (https://[www.planet.com](http://www.planet.com); Planet Team, 2017)^9^, after the Poiqu disaster that July. The central blue squiggle is the pathway of the landslide/GLOF/debris flow. The dotted white line is the Tibet/Nepal border in the vicinity of Kodari. The mosaic shows that landslides are far more numerous toward the south; these include landslides triggered by the Poiqu event, by the Gorkha earthquake (which Figure 1bshows are concentrated in the area toward the south), and possibly general monsoon-triggered landslides if the monsoon is stronger toward the south. The more southernly locations are also in the Lesser Himalaya (Figure S1), which is known to generate more landslides than the Greater Himalaya. (A-D) Time series of the development of river-blocking landslides. Panel B shows some minor landslides developed at or near the time of the Gorkha earthquake on April 25, 2015. Panel C shows no further major landslides in this area during the monsoon of 2015. Panel D then shows the development of massive landslides most likely at the time of the Poiqu disaster, and then landslide dammed lakes developed. The three main landslides are all sourced where the small earthquake landslides had formed. Panel D also exhibits distortions that are common in Google Earth imagery when landslides disturb the DEM used to orthorectify the images. See also Figure 12 and Figure 11 landslide number L4 for more information on these river-blocking landslides. Panels (E-H) show that the decade prior to the Gorkha earthquake had very little landslide activity, but then large landslides were generated by the Gorkha earthquakes. A year after the Poiqu event, further major reactivations occurred, presumably during the Poiqu event. H also shows that the river avulsed and a deposit of river sediment was laid down where the river had been. Landslides in panels G and H are also shown from the ground in Figure S12 panels C and E. Similar to the previous time series, Panels I-L shows that the decade before the Gorkha earthquake had little activity, and then minor landslides developed or were reactivated by the Gorkha earthquakes. Then widespread landsliding happened in presumed association with the Poiqu event. The photographs were taken by co-authors Jeffrey S. Kargel and Alina Karki during fieldwork.


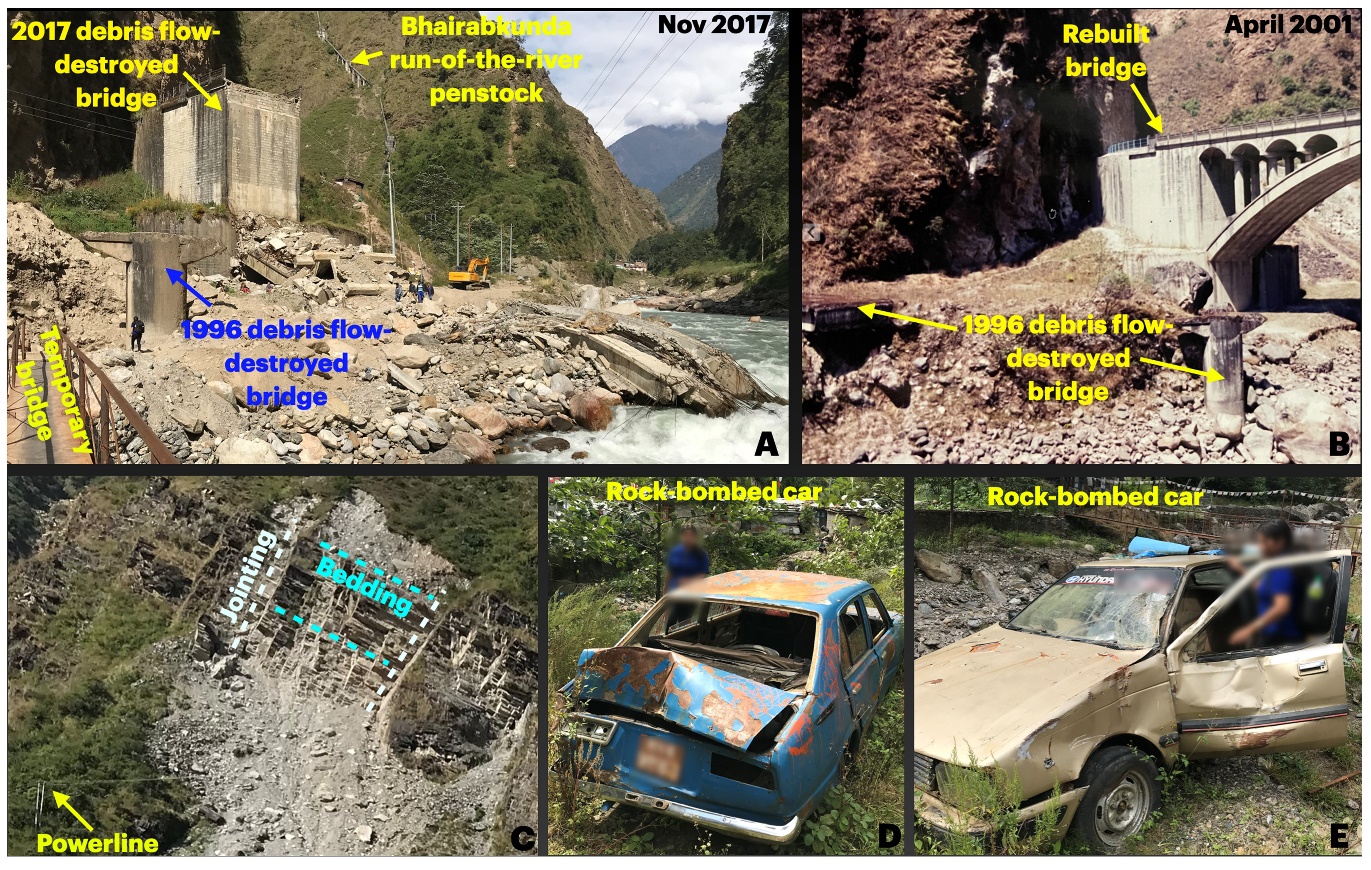


**Figure S4.** Additional examples of damaged infrastructure and property in the Upper Bhotekoshi valley due to other disasters in addition to the 2016 GLOF. The valley has a decades-long history of repeat events at the same localities. Bridges, the highway, hydroelectric power plants, and villages up and down the valley were damaged by the 2014 Jure landslide, the 2015 Gorkha earthquake, the 2016 Gongbatongsha GLOF, and multiple other landslides, including one in 2017. The rocks are prone to failure, and each big event conditions the next. (A) Remnants of a bridge at Larcha collapsed from a July 22, 1996 debris flow triggered by a landslide-dammed lake outburst of the Bhotekoshi River^7^. The bridge was replaced with a higher, more robust bridge, which then cracked in the 2015 earthquake, then was patched with metal supports; it survived the 2016 GLOF, but then was destroyed by a July 2017 rainfall-triggered landslide (<https://kathmandupost.com/national/2017/07/24/bridge-over-bhote-koshi-river-collapses-in-sindhupalchok>). In the 1996 event at the same location and with the same landslide source as in 2017, fifty-four people were killed. In the background is a penstock of the 3-MW Bhairabkunda run-of-the-river project, which was damaged on April 26, 2015 by a M6.9 aftershock a day after the Gorkha Earthquake mainshock. This project’s power lines were damaged in the 2014 Jure landslide, the 2015 Gorkha earthquake, the 2016 Gongbatongsha GLOF, and the 2017 landslide. For example, see: <https://kathmandupost.com/money/2014/08/03/landslide-hits-five-hydropower-projects>. The 2017 landslide also damaged the project’s headworks and tunnel. (B) Same area as Panel A in an April 2001 photo, showing the 1996-destroyed Larcha bridge and an improved replacement. (C) Power transmission lines from all of the upper valley’s hydropower projects have been repeatedly damaged almost every year, such as this example. (D and E) Several cars that were blocked from safe egress from the area due to the destroyed bridges were then also destroyed by rockfalls. See also Cowan (2021)^8^ for a description of repeated geological disasters in the valley. Photos by J. Kargel in Nov 2017, except panel B is from April 2001. The photographs were taken by co-authors Jeffrey S. Kargel and Alina Karki during fieldwork, 2017.

**Table S2.** Data used to derive the DEM of difference (DoD).

| **Situation** | **Data ID** |
| --- | --- |
| Pre Gorkha quakes | HMA_DEM8m_AT_20121224_0500_102001001FDA8A00_102001001F0E2100  HMA_DEM8m_AT_20150207_0505_105041001229DE00_105041001229DF00  HMA_DEM8m_AT_20150221_0515_103001003E1DCD00_103001003E6C4500 |
| Post Gorkha quakes | HMA_DEM8m_AT_20150525_0612_102001003DB53700_102001003D557D00 HMA_DEM8m_AT_20150602_0624_102001003DC6F100_102001003E440D00 HMA_DEM8m_AT_20151111_0512_103001004C0E1400_103001004C3BF900 HMA_DEM8m_AT_20151114_0506_1040010014461D00_10400100148CFE00  HMA_DEM8m_AT_20151114_0506_1040010014461D00_10400100148CFE00 HMA_DEM8m_AT_20151223_0522_10400100162FEB00_10400100163ADE00  HMA_DEM8m_AT_20151225_0715_10200100473A6400_10200100496C9000 |


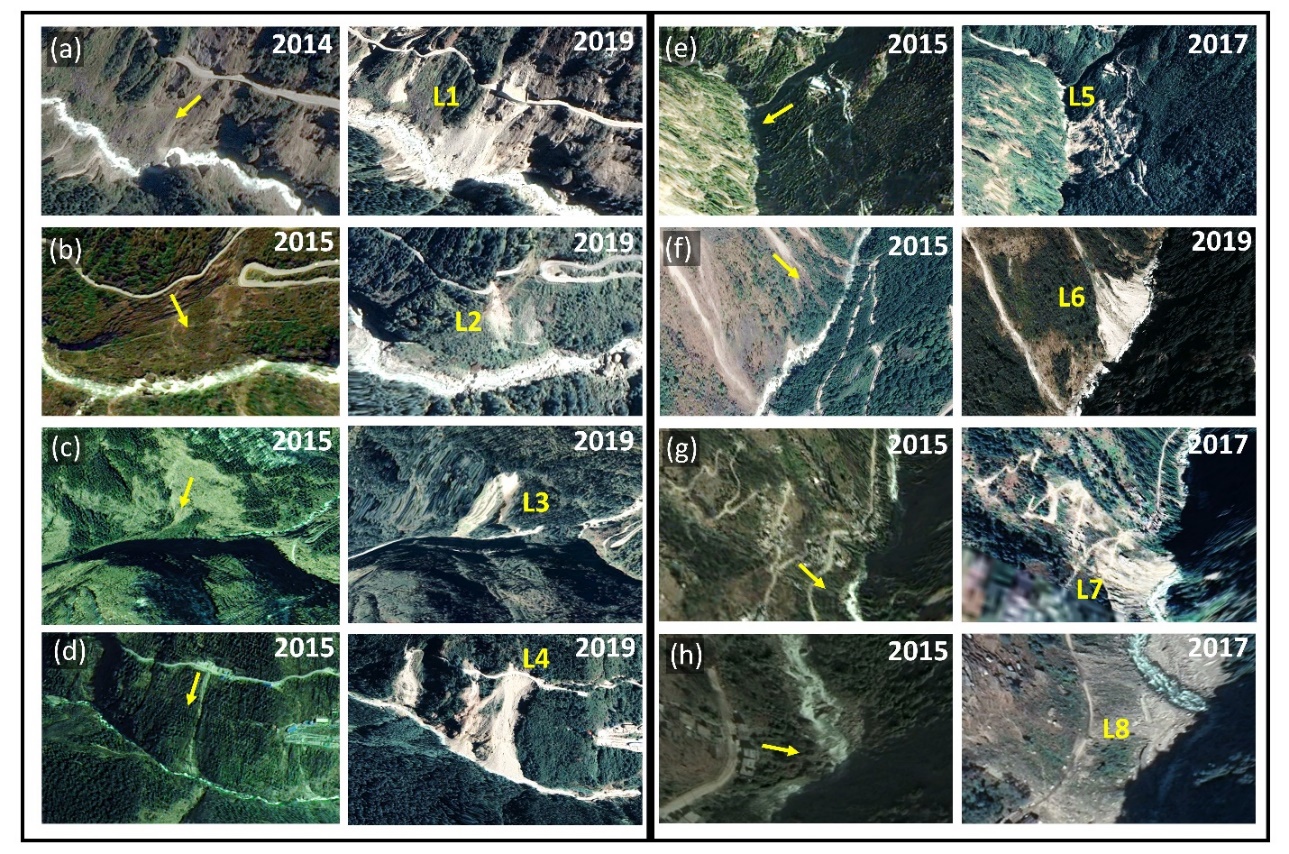


**Fig. S5.** Eight GLOF triggered landslides (L1 to L8) mapped in the valley from the Gongbatongsha Lake to the Bhotekoshi hydropower plant. Background: Pre- and Post-GLOF imageries from Google Earth (©CNES/Airbus Maxar Technologies).

**References:**

1. Roering, J. J., Kirchner, J. W., & Dietrich, W. E. (2005). Characterizing structural and lithologic controls on deep-seated landsliding: Implications for topographic relief and landscape evolution in the Oregon Coast Range, USA. Geological Society of America Bulletin, 117(5-6), 654-668.
2. Safaei, M., Omar, H., Huat, B. K., & Yousof, Z. B. (2012). Relationship between Lithology Factor and landslide occurrence based on Information Value (IV) and Frequency Ratio (FR) approaches–Case study in North of Iran. Electron J Geotech Eng, 17, 79-90.
3. Budathoki, R. (2016). Cause and Mechanism of 2014 Jure Rock Avalanche in Sindhupalchowk District, Central Nepal. PhD Dissertation, Tribhuvan University Central Department of Geology.
4. Karki, A., & Kargel, J. S. (2017). Lithology and Bedrock Geotechnical Properties in Controlling Rock and Ice Mass Movements in Mountain Cryosphere. In AGU Fall Meeting Abstracts (Vol. 2017, pp. NH41D-06).
5. Kargel, J. S., Karki, A., Haritashya, U. K., Shugar, D. H., & Harrison, S. (2017). Himalayan Lake-and River-Impacting Landslides and Ice Avalanches: Some So Deadly, Some No Problem. In AGU Fall Meeting Abstracts (Vol. 2017, pp. NH41D-07).
6. Dhital, M. R. (2015). Geology of the Nepal Himalaya: regional perspective of the classic collided orogen. Springer.
7. Oven, K. (2009). Landscape, livelihoods and risk: community vulnerability to landslides in Nepal (Doctoral dissertation, Durham University).
8. Cowan, S., 2021. The Araniko Highway conundrum. The Record, <https://www.recordnepal.com/the-araniko-highway-conundrum>
9. Planet Team: Planet Application Program Interface: In Space for Life on Earth, in, San Francisco, CA, 2017
